# Molecular and Phenotypic Evidence of a New Species of Genus *Esox* (Esocidae, Esociformes, Actinopterygii): The Southern Pike, *Esox flaviae*

**DOI:** 10.1371/journal.pone.0025218

**Published:** 2011-12-02

**Authors:** Livia Lucentini, Maria Elena Puletti, Claudia Ricciolini, Lilia Gigliarelli, Diego Fontaneto, Luisa Lanfaloni, Fabiana Bilò, Mauro Natali, Fausto Panara

**Affiliations:** 1 Dipartimento di Biologia Cellulare e Ambientale, Università degli Studi di Perugia, Perugia, Italy; 2 Division of Biology, Imperial College London, Silwood Park Campus, Berkshire, United Kingdom; 3 Veneto Agricoltura Viale dell'Università, Legnaro, Italy; 4 Provincia di Perugia, Via Angelucci, Perugia, Italy; University of Canterbury, New Zealand

## Abstract

We address the taxonomic position of the southern European individuals of pike, performing a series of tests and comparisons from morphology, DNA taxonomy and population genetics parameters, in order to support the hypothesis that two species of pike, and not only one, exist in Europe. A strong relationship emerged between a northern genotype supported by COI, Cytb, AFLP and specific fragments, and a phenotype with round spot skin colour pattern and a large number of scales in the lateral line, clearly separated from a southern genotype with other skin colour pattern and a low number of scales in the lateral line. DNA taxonomy, based on a coalescent approach (GMYC) from phylogenetic reconstructions on COI and Cytb together with AFLP admixture analysis, supported the existence of two independently evolving entities. Such differences are not simply due to geographic distances, as northern European samples are more similar to Canadian and Chinese samples than the southern Europe ones. Thus, given that the differences between the two groups of European pike are significant at the phenotypic, genotypic and geographical levels, we propose the identification of two pike species: the already known northern pike (*Esox lucius*) and the southern pike (*E. flaviae* n.sp.). The correct identification of these two lineages as independent species should give rise to a ban on the introduction of northern pikes in southern Europe for recreational fishing, due to potential problems of hybridisation.

## Introduction

Freshwater habitats support most ecosystem services, but their integrity is continuously affected by anthropogenic threats [1]–[3], as the introduction of non-native species and individuals for recreational fishing [4], [5]. These introductions may have a major impact on local diversity, as they are carried out by organised fishing associations, which re-stock local populations of target species using non-native animals. The impoverishment of phenotypic and genetic diversity is a known consequence of these activities in European salmonids [6], [7], where local populations can represent divergent lineages, potential endemic taxa [8], [9]. Actions should be taken to counteract extinction of endemic taxa and populations because of stocking with non-native individuals. Concerns on threatened fish other than salmonids are indeed present, and identification of divergent, endemic lineages should be considered urgently, especially for widespread species targeted by recreational fishing. Among those, the northern pike, *Esox lucius* Linnaeus, 1758, is extensively managed in Europe.

**Figure 1:**
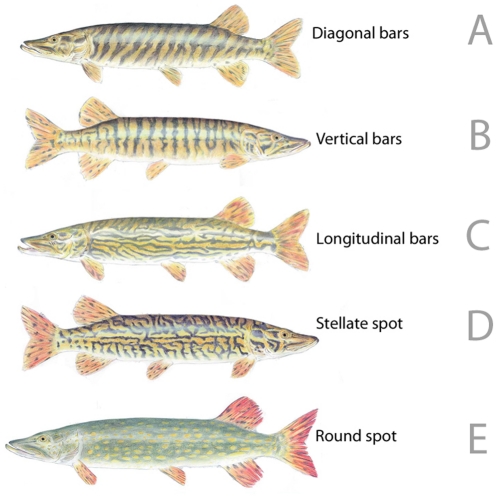
The five colour patterns recognized in European pikes.

The genus Esox is the only living genus in the family Esocidae, with five currently known species inhabiting North America, Europe and Eurasia. The phylogenetic relationships and biogeography of these species have been already studied [10]–[12], and the northern pike is the only native esocid in Europe (Text S1). Recent studies indicated a decline of southern populations [13]–[15], potentially due to its negative sensitivity to increasing water temperature [16], suggesting that this decline might be partly due to climate changes, as demonstrated for other species [17]. The negative effects of the contraction of these populations in Mediterranean countries have been managed through stocking programs with fries from northern Europe. This practice brought, as a consequence, the appearance of a phenotype, described as a yellow spot on a dark-coloured base (Figure 1), that was never seen earlier than 15 years ago in southern Europe [5]; [6] and that is the one described by Fickling [18] as with round/oval spots. To date, the question of whether this morphological difference between southern and northern European pikes suggests the existence of putative genetic separation of the two areas has not been investigated, even though the presence of distinct evolutionary entities in the European pike concerns biodiversity preservation, fish aquaculture and freshwater ecosystem management.

Here we explicitly test the hypothesis that the different phenotypes of the pike, geographically isolated in Europe, may represent two different evolutionary entities, in order to have reliable information for the management of wild populations of pikes in Europe. We analysed phenotypic and genetic differences sampling several individuals from different populations of European pike: as for the phenotype, we tested statistical differences in the general appearance of the skin pattern and in meristic characters as the number of scales in the lateral line. In order to identify the genetic signature of cryptic speciation we applied a coalescent based approach to mtDNA phylogeny; in order to support this signature with nuclear genetic data we evaluated the degree of historical admixture, testing overall genetic differences from amplified fragment-length polymorphism (AFLP). We also included information from populations outside Europe, present in the literature.

Our results strongly support the existence of two distinct lineages: thus, we here describe a new species of pike and provide phenotypic and genetic features to identify it unambiguously. As a consequence, we recommend stopping the stocking of pike in southern Europe using northern pike from other European countries, as this could greatly impact the survival of this newly discovered species in its native range.

## Materials and Methods

The work performed during the analyses carried out for this manuscript is consistent with National regulations and indications of the Ethics Committee of the University of Perugia. Approval by Ethics Committee was not necessary given the nature of the data collected (counts of lateral line scales, photos and conservative specimens) and the method of data recovery, without any animal suffering. In fact, all the animals were anaesthetized, samples for DNA analyses were collected with non invasive techniques, and animals were all released in the same sampling site, after recovery in freshwater tanks. In such cases the Ethics Committee waives the need for consent. The only dead individual is the holotype, one of the spawners used in supportive breeding, accidentally killed during artificial spawning activity.

### Sample collection

Sampling was not invasive: pictures of the right side of animals were collected for the morphological analyses, and 10 mg of caudal fin or a few scales were collected for the genetic analyses. The right side of 1306 mature individuals, longer than 30 cm was photographed by means of a digital photo camera. These samples were collected in three areas in Europe: continental Europe (Holland, Switzerland, Czech Republic, Hungary and Sweden), Northern Italy (Po basin, 11 populations) and Central Italy (Tevere basin, 4 populations) (Text S2).

### Phenotypic analyses

The number of scales in the lateral line is a taxonomically informative meristic character that can provide clear-cut separation between populations of the northern and southern pike. The number of scales was counted for each individual. Counts were performed twice in different days, in order to check for consistency between measurements and no disagreements were found between repeated counts. Differences in the number of scales between the two potential groups of pike were tested by generalised linear models (GLM), implementing a quasi-Poisson distribution for count data, accounting for overdispersion [19]. GLM were performed in R 2.12.0 (R Development Core Team 2011).

The skin colour pattern is a qualitative character that can provide another useful tool for the discrimination of the two groups of pike in Europe. Presence/absence of such patterns can be analysed quantitatively to provide statistical support for the hypothesis of two separate entities. Five basic skin colour patterns were identified and noted, according to Fickling [18] with some modifications: we considered round spot (RS), stellate spot (SS), diagonal bars (DB), longitudinal bars (LB) and vertical bars (VB) (Figure 1). Differences in the presence of the five skin colour patterns in the two groups were tested by GLM in R, implementing a quasi-Poisson distribution.

### Genetic analyses

All individuals were sampled non-invasively and were immediately reintroduced in the environment of origin. Genomic DNA was extracted and quantified as previously reported [20], [21] on 374 samples belonging to the four identified areas.

#### Independent lineages of mtDNA

DNA taxonomy, providing species delimitation based on the topology of gene trees, is becoming a powerful tool to help taxonomists support their decisions [20], [22]. We used two mtDNA loci, Cytochrome Oxidase Subunit I (COI) and Cytochrome b (Cytb), to reconstruct phylogenetic relationships and test for statistical evidence of two independently evolving entities using a coalescent-based approach. COI and Cytb are known to be useful in barcoding and DNA taxonomy in fishes [23]–[31]; moreover, these proteins might be related to oxygen availability and, thus, to water temperature, which is one of the main physical characters distinguishing northern and southern European freshwaters.

No nuclear copies of the COI and Cytb region have been reported in fishes; nevertheless, because preliminary PCR amplifications with Ivanova et al primers [32] displayed more than one band in electrophoretic visualization, we performed PCRs with more specific primers. COI amplicons of 651 bp were thus obtained using specifically designed primers F-COI-El GTGGCAATCACACGCTG and R-COI-El CGGGTGTCCGAAGAATC. For Cytb, amplicons of 1079 bp were obtained using specifically designed primers cytbf TCGGACTCTACCAAAACCAA and cytbr GTTCAACGGGTATTCCTCCA. PCR reactions were performed with Ready-to-go-DNA-PCR-Beads (GE) with the protocol described by Lucentini et al [14]. PCR products were purified and sequenced in forward and reverse directions as reported by Lucentini and colleagues [33].

Sequence identities were evaluated by blasting procedure (http://www.ncbi.nlm.nih.gov/BLAST/). Sequences were aligned and edited using MEGA 4.0. Moreover, we included in our dataset all COI and Cytb sequences of the genus *Esox* available from GenBank on May 20^th^, 2011 (Table 1). These sequences were used to widen the geographic coverage of the sample of the northern pike (including areas as Canada and China, geographically distant from our European samples), and to have outgroup information to root the phylogenetic trees.

**Table 1:** COI and Cytb datasets.

COI			Cyt b		
GenBank	Species	Haplotype Code	GenBank	Species	Haplotype Code
EU524612	*Esox niger*	-	AY497428-30	*Esox americanus*	-
EU524602	*Esox masquinongy*	-	AY497433-36	*Esox americanus*	-
EU524598	*Esox masquinongy*	-	JN190460	*Esox flaviae*	Cytbhap3
EU524577	*Esox americanus*	-	JN190463	*Esox flaviae*	Cytbhap6
EU524573	*Esox americanus*	-	JN190464	*Esox flaviae*	Cytbhap7
HM563707	*Esox flaviae*	COIhap 20	JN190465	*Esox flaviae*	Cytbhap8
HM563706	*Esox flaviae*	COIhap 19	JN190471	*Esox flaviae*	Cytbhap14
HM563705	*Esox flaviae*	COIhap 18	JN190472	*Esox flaviae*	Cytbhap15
HM563704	*Esox flaviae*	COIhap 17	JN190476	*Esox flaviae*	Cytbhap19
HM563703	*Esox flaviae*	COIhap 16	JN190477	*Esox flaviae*	Cytbhap20
HM563700	*Esox flaviae*	COIhap 13	JN190481	*Esox flaviae*	Cytbhap24
HM563698	*Esox flaviae*	COIhap 11	JN190482	*Esox flaviae*	Cytbhap25
HM563697	*Esox flaviae*	COIhap 10	JN190484	*Esox flaviae*	Cytbhap27
HM563696	*Esox flaviae*	COIhap 9	JN190485	*Esox flaviae*	Cytbhap28
HM563695	*Esox flaviae*	COIhap 8	JN190486	*Esox flaviae*	Cytbhap29
HM563694	*Esox flaviae*	COIhap 7	JN190487	*Esox flaviae*	Cytbhap30
HM563692	*Esox flaviae*	COIhap 5	FJ425091-95	*Esox lucius*	-
HM563691	*Esox flaviae*	COIhap 4	FJ425097	*Esox lucius*	-
HM563688	*Esox flaviae*	COIhap 1	HM177469-70	*Esox lucius*	-
EU524592	*Esox lucius*		HM592193	*Esox lucius*	-
EU524586	*Esox lucius*		JN190458	*Esox lucius*	Cytbhap 1
EU524578	*Esox lucius*		JN190459	*Esox lucius*	Cytbhap 2
HQ600729	*Esox lucius*		JN190461	*Esox lucius*	Cytbhap 4
FJ896108	*Esox lucius*		JN190462	*Esox lucius*	Cytbhap 5
HM563702	*Esox lucius*	COIhap 15	JN190466	*Esox lucius*	Cytbhap 9
HM563701	*Esox lucius*	COIhap 14	JN190467	*Esox lucius*	Cytbhap 10
HM563699	*Esox lucius*	COIhap 12	JN190468	*Esox lucius*	Cytbhap 11
HM563693	*Esox lucius*	COIhap 6	JN190469	*Esox lucius*	Cytbhap 12
HM563690	*Esox lucius*	COIhap 3	JN190470	*Esox lucius*	Cytbhap 13
HM563689	*Esox lucius*	COIhap 2	JN190473	*Esox lucius*	Cytbhap 16
			JN190474	*Esox lucius*	Cytbhap 17
			JN190475	*Esox lucius*	Cytbhap 18
			JN190478	*Esox lucius*	Cytbhap 21
			JN190479	*Esox lucius*	Cytbhap 22
			JN190480	*Esox lucius*	Cytbhap 23
			JN190483	*Esox lucius*	Cytbhap 26
			AY497437	*Esox niger*	-
			AY497442	*Esox reicherti*	-
			AY497444	*Esox reicherti*	-

Haplotype codes are given for the individuals newly sequenced for this study, whereas no code has been given to sequences downloaded from GenBank.

Phylogenetic analyses were performed separately for each locus. The COI dataset comprised 30 haplotypes (20 from the present study and 10 from GenBank) with 651 bp. The Cytb dataset comprised 46 haplotypes (30 from the present study and 16 from GenBank) with 998 bp (Table 1). ModelGenerator 0.85 [34] was used to select the best evolutionary models for the phylogenetic reconstructions, which resulted HKY+G for both datasets, according to AIC and BIC. Bayesian inference analyses were run in MrBayes 3.1.2 [35] for 3 million generations with two parallel searches. Maximum Likelihood (ML) reconstructions were performed using PhyML 3.0 [36], with 100 bootstrap replicates to provide support for the branching pattern.

The generalised mixed Yule coalescent (GMYC) model [37], [38] was used to detect independent evolutionary lineages, evidence of distinct species from the topology of the tree. We used the output of the Bayesian inference as a starting tree for both loci, including only the dataset from *Esox lucius*, rooted with the closest of the other *Esox* (all other *Esox* for COI and only *E. reicherti* for Cytb). We then converted the tree into an ultrametric tree testing for the most appropriate smoothing parameter using r8s 1.71 [39]. The GMYC protocol considers a null model that the sample of individuals derives from a single evolutionary entity following a single coalescent process. The alternative is that the sample represents several independently evolving entities: i.e. selection and drift operate independently in different entities. In this case, coalescence occurs separately in different entities, leading over time to the appearance of discrete genetic clusters, separated from each other by longer internal branches. The method uses a maximum likelihood approach to optimize the shift in the branching patterns of the gene tree from interspecific branches (Yule model) to intraspecific branches (neutral coalescent), and thereby identifies clusters of sequences corresponding to independently evolving entities. It does this by optimizing the maximum likelihood value of a threshold such that nodes before the threshold are identified as species diversification events, while branches beyond the threshold are clusters following coalescent processes. We used a Likelihood Ratio test to support the scenario that the result of this Maximum Likelihood threshold is a better explanation of the tree topology than the null model (i.e. the sample of sequences belongs to a single population obeying a single coalescent process). Models were fitted in R 2.12.0 with the package splits (http://splits.r-forge.r-project.org).

Uncorrected pairwise distances between haplotypes were calculated in R, package ape 2.6–1 [40]. To assess distinctiveness level of European populations and their contribution to the overall genetic species variability, differences between European pike and congeneric from other continents were analysed using indices of divergence from population genetics. We identified variable and parsimony informative sites, translation of nucleotide sequences, pairwise genetic distances, nucleotide base composition, transition/transversion ratios and the Tajima's Neutrality Test (TNT), using MEGA4.0. Moreover, we calculated F_st_ values between taxa using ARLEQUIN3.5 [41].

#### AFLP analysis

AFLP analysis produces reliable multilocus fingerprints of complex genomes without any previous species-specific information [42] and it has been already used to distinguish morpho-species and phenotypes in fishes [43]–[44]. Thus, AFLPs can screen the entire genome to search for genotype-phenotype relationships [45]. As briefly reviewed by Papa and colleagues [46] several analytical improvements have been made in recent years, in particular the use of fluorescence labelled primers and the resolution of the band pattern by means of automatic capillary sequencers instead of polyacrylamide gel. This has greatly reduced the time and cost, while improving pattern reproducibility [46]. The quantitative information for single individuals is essentially dependent on the specific endonucleases to be used for the restriction step; *Eco*RI/*Taq*I combinations in genomes of high complexity have greatly improved polymorphism and profile quality if compared with *Eco*RI/*Mse*I combinations [46]–[48].

The present research applies, for the first time, an extensive application of ten different *Eco*RI/*Taq*I AFLPs combinations in European pike. We analysed the overall patterns of nuclear genetic variation among northern pike populations using a fluorescent AFLP procedure [46] with just a few modifications: 500 ng of genomic DNA were restricted with *Taq*I for 1.5 hour at 65°C and, subsequently, for 2 hours with *Eco*RI at 37°C. The selective nucleotides were AAG/AAC, AGC/AAC, AGC/AAG, AAC/AAC, AGT/ACG, AAC/AAG, AAG/AAG, AAG/ACG, AGC/ACG, AGT/AAC. One micro litre of a mixture made of FAM, PET, NED, or VIC 5′-labelled amplicons and 0.3 µl of GeneScan-500 LIZ Size Standard were added to 18.7 µl of Hi-Di Formamide, denaturised and run on an ABIPRISM310. The reproducibility of AFLP markers was tested as suggested by Papa et al [46] and by analysing different extractions per sample, by repeating the AFLP assay with twenty random samples and using the chosen selective primer pairs. Fragments were analysed through GeneMapper 5.0, eliminating bands under 150 bp to reduce the risk of homoplasy [44]. Statistical analyses were both band-based (BB) and allele frequency-based (FB) and conducted as already assessed (see Table 1 in Bonin et al. [49]).

F_st_ values between groups of individuals defined by the five categories of skin colour patter were computed with AFLP-SURV [50]. Statistical significance of the differences in F_st_ values between northern (round spot) and southern skin colour patter (stellate spot, vertical, diagonal and longitudinal bars) was estimated using a permutational multivariate analysis of variance using distance matrices with the *adonis* function in R, package vegan 1.17-4 [51].

In order to independently verify if the skin colour pattern assignment was related to AFLP-derived genotypes, STRUCTURE 2.3.3 [52] was used to implement the non-spatial Bayesian clustering methods using the AFLP data aggregated by skin colour pattern. The applied algorithm divides sampled individuals into a number of clusters (K) and tests for the most likely value of K, given the data, and maximises log Pr(X|K) given the data (X); we performed the test setting K from 1 to 20. Twenty independent runs for each K between 1 and 20 using the admixture model and correlated allele frequencies were made. Exploratory structure runs demonstrated that a burn-in period of 100,000 steps, followed by 500,000 steps of data collection, ensure the convergence of the MCMC. Moreover, a Mantel test was performed between the matrix of F_st_ values between the analysed populations and the geographical distances between them, in order to identify geographic signatures of genetic diversification in Europe. Geographical distances were specified as the matrix of all paired kilometric distances, estimated from angular distances between paired coordinates.

#### Identification of specific polymorphic AFLP fragments

In order to identify a quick and reliable genetic marker for the identification of the two species, thirty-two AFLP polymorphic clearly visible and distinct bands were selected. Following the Bensch et al. [53] protocol, these thirty-two bands were processed and, finally, two bands were identified and positively sequenced: band 9 and 24. Thus, additional primers were designed (9FW: CAGTTGTAAGGCCCAGGAAG 9RV: GGAAATACGTTGTGGAACTGC; 24extFW: GATCTCTGGACCATTTGGAC 24extRV: TGGCTACATGCGACATCAG) and used to amplify these bands in additional 71 individuals. Results were controlled verifying the presence/absence of each AFLP band correlating with the individual phenotype.

### Nomenclature Acts

The electronic version of this document does not represent a published work according to the International Code of Zoological Nomenclature (ICZN), and hence the nomenclatural acts contained in the electronic version are not available under that Code from the electronic edition. Therefore, a separate edition of this document was produced by a method that assures numerous identical and durable copies, and those copies were simultaneously obtainable (from the publication date noted on the first page of this article) for the purpose of providing a public and permanent scientific record, in accordance with Article 8.1 of the Code. The separate print-only edition is available on request from PLoS by sending a request to PLoS ONE, 1160 Battery Street, Suite 100, San Francisco, CA 94111, USA along with a check for $10 (to cover printing and postage) payable to “Public Library of Science”.

In addition, this published work and the nomenclatural acts it contains have been registered in ZooBank, the proposed online registration system for the ICZN. The ZooBank LSIDs (Life Science Identifiers) can be resolved and the associated information viewed through any standard web browser by appending the LSID to the prefix “http://zoobank.org/”. The LSID for this publication is: urn:lsid:zoobank.org:pub:B826CDA6-01B6-4D68-B998-5EFB7D70A9A9.

## Results and Discussion

### Phenotypic differences

**Figure 2:**
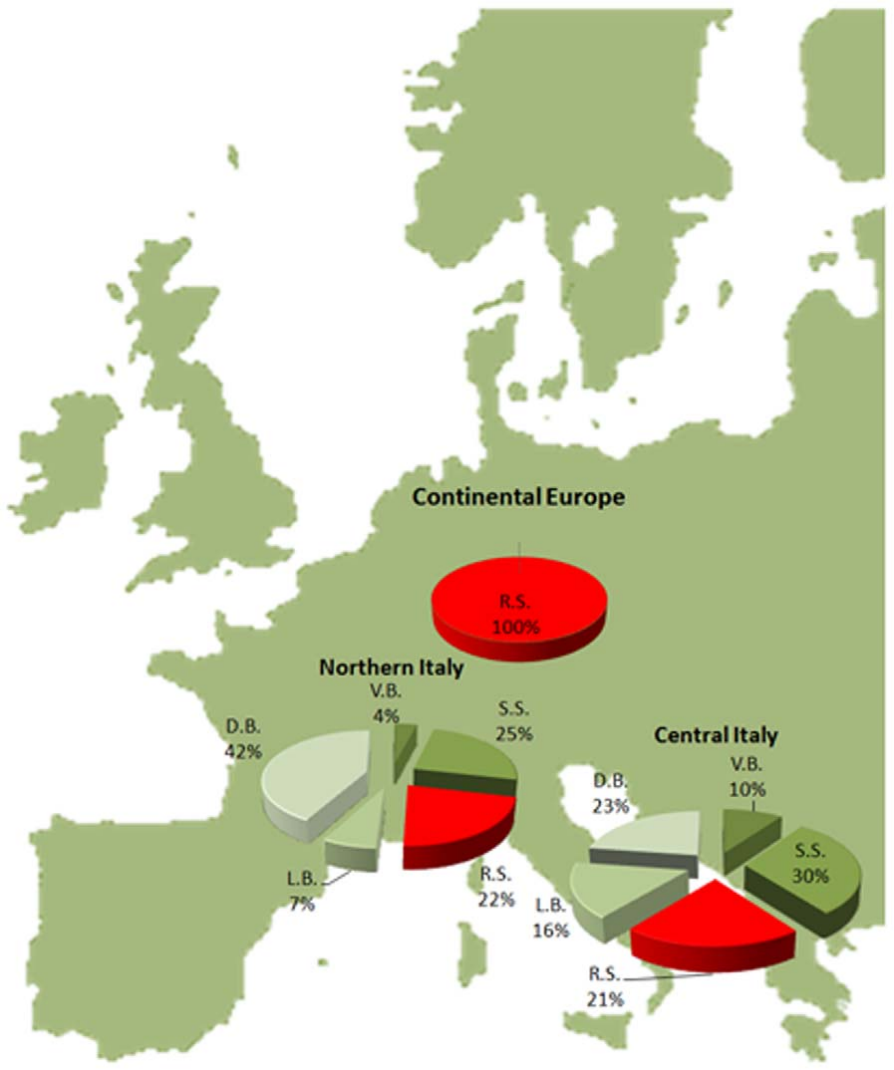
Distribution of the five colour patterns shown by pike in Europe. Round spot (RS); stellate spot (SS), diagonal bars (D.B.), longitudinal bars (L.B.) and vertical bars (V.B.)

The analysis of the skin colour pattern of European pikes showed a recognizable distribution of phenotypes around Europe: in Central-West and North-East Europe the round-spot phenotype was dominant (Figure 2) whereas in Italy it seems confined just to some populations; on the other hand, the other four skin colour patterns (stellate spot, diagonal, longitudinal and vertical bars) are particularly diffused in this area. The distribution of the round spot skin pattern, together with the fact that this phenotype was never seen in Italy before the start of the re-stocking activities [Natali M., personal communication], strongly suggests that its appearance in Italy could be due to artificial transportation.

**Figure 3:**
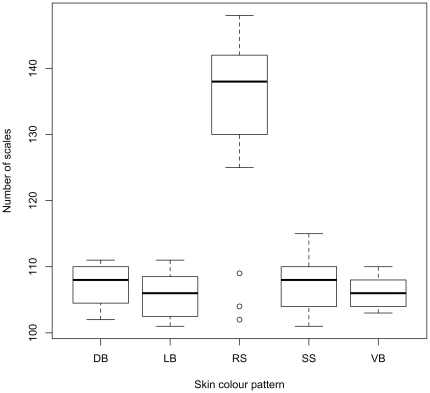
Boxplot of the distribution of the number of scales vs skin colour pattern. Boxplot of the distribution of the number of scales in the lateral line for the individuals belonging to the five skin colour pattern (round spot (RS), stellate spot (SS), diagonal bars (DB), longitudinal bars (LB) and vertical bars (VB), refer to Figure 1). The horizontal thick bar represents the median value, the box includes 50% of the data, the whiskers get to the most distant value within the 95% of the distribution, and dots mark outliers, outside the 95% of the distribution.

The number of lateral line scales in our European samples ranged from 101 to 148, and almost completely overlaps with the numbers reported in the literature across all the palearctic range of the northern pike: between 103 and 148 [54]. The number of scales did not show significant differences among the four southern phenotypes (diagonal, vertical and longitudinal bars, and stellate spot; GLM: all p>0.4), whereas the difference was highly significant between these four phenotypes and the round spot skin pattern (GLM: t = 11.9, p<0.0001). Independently of the geographic area of origin, samples showing the four southern skin colour patterns have a significantly lower number (GLM: t = 20.8, p<0.0001) of lateral line scales, ranging from 101 to 115, than those showing the round-spot phenotype, whose scales ranged generally from 125 to 148 (Text S3). The number of scales supports a clear distinction between the two groups, almost without any overlap. Interestingly, three individuals with a round-spot phenotype had a lower number of scales, 102, 104 and 109, typical of the other skin colour patterns. All these individuals came from one population in Lake Maggiore, where 3 different skin colour phenotypes and a number of scales from 102 to 147 were found out of only 9 analysed individuals (Figure 3). It is possible that northern pikes introduced in this area from northern Europe hybridised with the local populations, producing hybrids with northern skin colour pattern and southern number of scales. Hybridisation in fishes is a common event, both in closely related species and even between non co-generic species (see Epifanio and Nielsen [55] for a review). In the genus *Esox*, hybridisation between co-occurring species is possible and has been demonstrated in North America, where different species have overlapping ranges [56].

### Discriminating lineages with mtDNA

**Figure 4:**
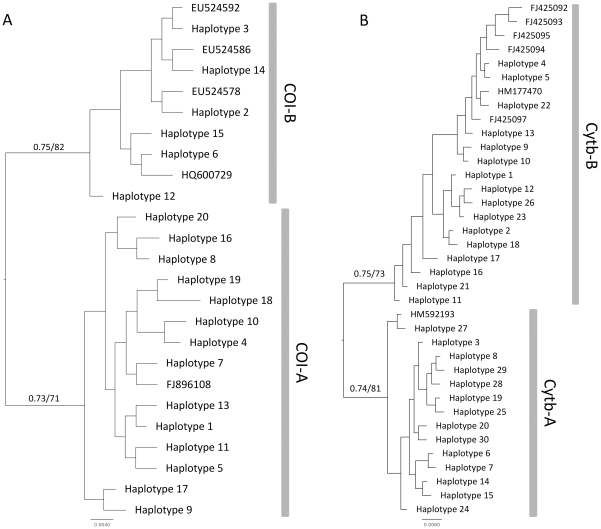
Consensus tree. Consensus tree of 20,000 sampled trees from the Bayesian inference search on the (A) COI and (B) Cytb alignment of all haplotypes of the pike using the GTR+I+G model for nucleotide substitution in both cases. COI-B and Cytb-B refer to the nominal species *Esox lucius s.s.*, whereas COI-A and Cytb-A refer to the new species, *E. flaviae* n.sp., described later. Averaged branch lengths are proportional to substitutions per site under the used model. Bayesian posterior probabilities/Bootstrap support values of the ML analysis are provided above branches. Vertical grey bars identify the clusters representing independently evolving entities according to the GMYC model.

The 651 bp COI-region and the 998 bp Cytb-sequence were successfully sequenced and could be aligned unambiguously for the entire sample set. From our European dataset, 20 haplotypes were identified for COI, whereas 30 were identified for Cytb (Table 1). Phylogenetic reconstructions for both loci, including additional haplotypes from GenBank, provided evidence of two well-supported groups (Figure 4). The two groups received significant support from the GMYC model, which provided evidence for two independently evolving entities, equivalent to two distinct species, in the northern pike (Table 2).

**Table 2:** Summary statistics of the GMYC model for COI and Cytb.

	COI	Cytb
likelihood of null model	66.59	128.74
maximum likelihood (ML) of GMYC model	71.07	133.25
likelihood ratio (LR)	8.94	9.01
result of LR test	0.029	0.028
number of ML entities	2	2

For COI, one potential species, here called COI-A, included 15 haplotypes, 11 from southern Europe and 4 from the Danube river; the other potential species, COI-B, included 9 haplotypes, 6 from southern, central, northern and eastern Europe, 1 from Greece and 3 from Canada. Thus, according to the two COI groups, some European pikes are more similar to Canadian pikes than to most Italian ones. We can argue that for COI, pikes of the COI-B clade are native to all the palearctic, except for Italy, where its occurrence can be due to introduction for recreational fishing, whereas pikes of the COI-A clade are native only in Italy with some individuals in the Danube river. The sample from Greece can be regarded as geographical dubious, because autoctony of the northern pike in this country is questionable [57], [58]. COI-B is the nominal species *Esox lucius*, whereas COI-A is a different species, which will be described later. Percentages of uncorrected genetic differences within each group were less than 1%, and from 1.1% to 2.6% between them (Table 3). Both within-species and between-species differences in other species of *Esox* have generally higher values (Table 3). Nevertheless, these values are congruent with those already reported on other freshwater fishes at both intra-specific and inter-specific-level. In fact, in Canadian fishes values below 1% within species and between 0% and 19.33% between species within genus have been found for COI [59]. The values we report for *Esox* are expected to be lower, as we used uncorrected distances, instead of the K2P model used for Canadian fishes; thus, comparable values with our more conservative approach in computing genetic distances provide even stronger support for the significant distances between the two species of pike in Europe. Moreover, COI is not able to resolve the species complex *E. niger* – *E. americanus*: *E. americanus* was paraphyletic, with *E. niger* nested within its genealogy [56], [59], suggesting that a high degree of introgression may have happened for these two species.

**Table 3:** Summary of the sequence divergence, transition/transversion ratios (Ti/Tv), number of variable sites (Vs) and number of parsimony-informative sites (Ps) between and within haplogroups.

Haplogroups	Average Divergence (range)	Ti/Tv 1st	Ti/Tv 2nd	Ti/Tv 3rd	Ti/Tv all	Vs	Ps
**Between multiple haplogroups**							
COI-A vs COI-B	1.80% (1.08–2.61)	6.15	220.62	0.00	6.64	23	0.03
**Within haplogroups**							
COI-A	0.42% (0.15–0.92)	222.59	0.00	2.93	1.28	15	0.02
COI-B	0.47% (0.15–0.77)	0.51	0.58	135.35	366.79	7	0.01
**Between multiple haplogroups**							
Cytb-A vs Cytb-B	2.19% (1.51–2.72)	348.87	317.87	20.85	24.55	40	0.04
**Within haplogroups**							
Cytb-A	0.34% (0.10–0.71)	321.05	11.66	21.46	260.96	13	0.01
Cytb-B	0.64% (0.10–1.41)	455.84	0.56	5.93	6.95	20	0.02

The overall transition/transversion bias is R = [A*G*k1+T*C*k2]/[(A+G)*(T+C)]. All positions containing gaps and missing data were removed from the dataset (Complete-deletion option). The Ti/Tv ratios are presented for 1st, 2nd and 3rd codon positions, and also summarized across all codon positions.

Non-synonymous substitutions in the COI sequence occurred in 24 amino acids out of 217; 11 of them occurred only in COI-A, 5 only in COI-B, 5 in both groups, and only 3 were completely discriminating the two groups. The nucleotide sequences composition across all haplotypes in the *E. lucius* species complex was moderately A+T rich (54.9%), with overlapping values between the two COI groups (COI-A: range 55.30–55.91%, average 55.73%; COI-B: range 55.45–55.76%, average 55.63%. These values are comparable with those of other esocids (55.3% for *E. masquinongy*; 55.8% for *E. americanus*). Tajima's test for neutrality underlined a p_s_ across *Esox* species of 0.23, between the *E. lucius* species complex and *E. americanus* of 0.17, whereas it was 0.15 between the *E. lucius* species complex and *E. masquinongy*. A value of 0.04 was found between the two COI groups within the *E. lucius* species complex. F_st_ values between species ranged from 0.93 (*E. lucius/E. americanus-E. masquinongy*) to 0.99 (*E. masquinongy/E. niger*), whereas F_st_ value between COI-A and COI-B was lower, only 0.57. This value, though lower than those obtained between other *Esox* species, is still high and greater than 0.25, i.e. the value that, according to Hartl and Clark [60], denotes a very great differentiation between isolated populations, which again may suggest the existence of distinct species.

**Figure 5:**
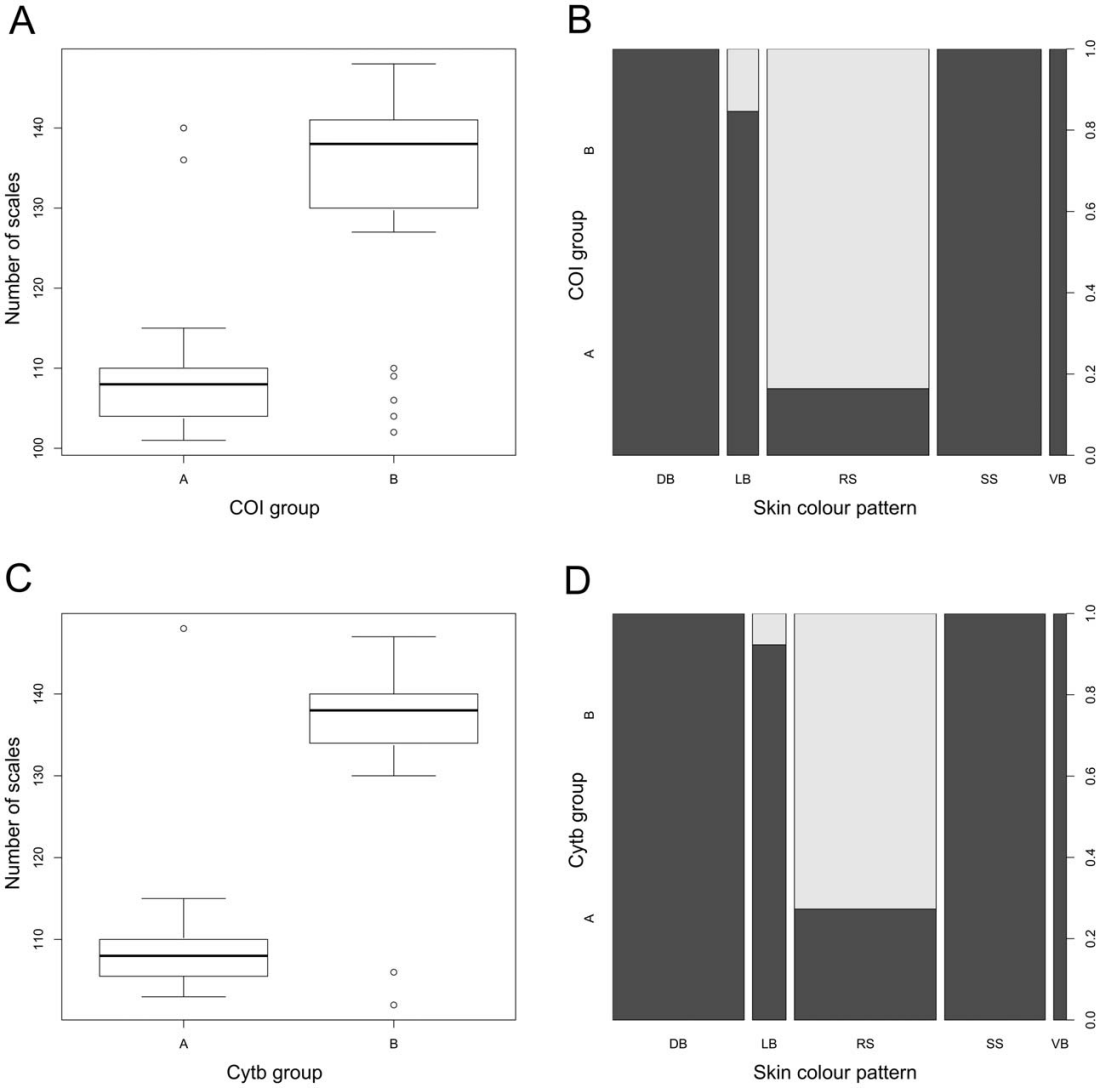
Boxplot of the distribution of the number of scales and skin colour pattern vs COI and Cytb lineages. **A.** Boxplot of the distribution of the number of scales in the lateral line for the individuals belonging to COI-A and COI-B mtDNA lineages. The horizontal thick bar represents the median value, the box includes 50% of the data, the whiskers get to the most distant value within the 95% of the distribution, and dots mark outliers, outside the 95% of the distribution. **B.** Proportion of individuals belonging to COI-A (dark grey) and COI-B (light grey) mtDNA lineages for each of the five skin colour pattern (round spot (RS), stellate spot (SS), diagonal bars (DB), longitudinal bars (LB) and vertical bars (VB), refer to Figure 1). **C.** Boxplot of the distribution of the number of scales in the lateral line for the individuals belonging to Cytb-A and Cytb-B mtDNA lineages. The horizontal thick bar represents the median value, the box includes 50% of the data, the whiskers get to the most distant value within the 95% of the distribution, and dots mark outliers, outside the 95% of the distribution. **D.** Proportion of individuals belonging to Cytb-A (dark grey) and Cytb-B (light grey) mtDNA lineages for each of the five skin colour pattern (round spot (RS), stellate spot (SS), diagonal bars (DB), longitudinal bars (LB) and vertical bars (VB), refer to Figure 1).

The two putative species had a clear scenario in their skin colour pattern: 90.5% of the individuals in COI-A had southern pattern (stellate spot, diagonal, longitudinal or vertical bars), and 96.5% of the individuals in COI-B had the round spot pattern (Figure 5A). The number of scales in the lateral line was significantly different in the two COI groups (GLM: t = 9.9, p<0.0001), with COI-A mostly having less than 115 scales and COI-B more than 125. The three individuals of COI-A with more than 125 scales all came from the Danube river, whereas 4 of the individuals of COI-B with less than 115 scales came from Maggiore lake and 1 from Chiusi lake (Figure 5B).

For Cytb, one potential species, here called Cytb-A, included 15 haplotypes, all from southern Europe, except for one individual from the Danube river, which shared haplotype 3 with many other Italian individuals; the other potential species, Cytb-B, included 22 haplotypes, 16 from southern, central, northern and eastern Europe, and 6 from China. Thus, in a similar scenario to the one for COI, according to the two Cytb groups, some European pikes are more similar to Chinese pikes than to most Italian ones. We can argue that also for Cytb, pikes of the Cytb-B clade are native to the palearctic, except for Italy, and are indeed the nominal species *E. lucius*, whereas pikes of the Cytb-A clade are native only in Italy, and, again, also present in the Danube river. Cytb-A represents a new species, described later. Percentages of uncorrected genetic differences within each group were less than 1.5%, and from 1.5% to 2.7% between them (Table 3). Both within-species and between-species differences in other species of *Esox* have generally higher values (Table 3): even the differences between the two subspecies of *E. americanus* are above 3%. On the other hand, also for Cytb, *E. niger* falls within *E. americanus*.

Non-synonymous substitutions in the Cytb sequence occurred in 9 amino acids out of 332; 2 of them occurred only in Cytb-A, 5 only in Cytb-B, 2 in both groups, and none was completely discriminating the two groups. The nucleotide sequences composition in *Esox* is moderately A+T rich, with very similar values for Cytb-A (55.85%) and Cytb-B (55.96%); slightly lower than in the other species (*E. americanus*: 58.47%, *E. reichertii*: 57.56%). Tajima's test for neutrality underlined a p_s_ across *Esox* species of 0.23, between the *E. lucius* species complex and *E. americanus* of 0.20, whereas it was 0.12 between the *E. lucius* species complex and *E. reichertii*. A value of 0.05 was found between the two Cytb groups within the *E. lucius* species complex. F_st_ values across *Esox* species of 0.90, between the *E. lucius* species complex and *E. americanus* of 0.94, whereas it was 0.95 between the *E. lucius* species complex and *E. reichertii*. A value of 0.79 was found between the two Cytb groups within the *E. lucius* species complex. This value, though lower than those obtained between other *Esox* species, is still high and greater than 0.25, i.e. the value that, according to Hartl and Clark [60], denotes a very great differentiation between isolated populations, which again may suggest the existence of distinct species.

The two putative species had a clear scenario in their skin colour pattern: 87.7% of the individuals in Cytb-A had southern pattern, and 97.5% of the individuals in Cytb-B had the round spot pattern (Figure 5C). The number of scales in the lateral line was significantly different in the two Cytb groups (GLM: t = 10.3, p<0.0001), with Cytb-A mostly having less than 115 scales and Cytb-B more than 125. One individual of Cytb-A with more than 125 scales came from the Danube river, whereas the two individuals of Cytb-B with less than 115 scales came one from Maggiore lake and one from Chiusi lake (Figure 5D). This scenario is very similar to the one described for COI groups. Actually, COI and Cytb groups overlapped almost completely: all individuals belonging to COI-A group were in Cytb-A, whereas only two individuals had COI-B and Cytb-A, one from the Danube and one from the Bacchiglione river.

### AFLP

The analysed markers provided high percentages of polymorphism (Table 4). F_st_ values for comparison between different skin colour patterns (Table 5) suggest a dual separation of samples, with very low F_st_ values between the four southern phenotypes (stellate spot, longitudinal, vertical and diagonal bars), and F_st_ values higher than 0.16 between round spot pattern and the other skin colour pattern. The differences between the two groups are strong (adonis: R^2^ = 0.99, p = 0.05).

**Table 4:** Primer combination, combination code and selective nucleotides used, together with the total number of fragments and of polymorphic bands for each combination.

Combination code	Selective nucleotides	Total number of fragments per primer combination	Number of polymorphic bands per primer combination	% of polymorphisms
A	AAG/AAC	47	26	55%
B	AGC/AAC	51	22	43%
C	AGC/AAG	50	26	52%
D	AAC/AAC	58	22	38%
E	AGT/ACG	43	31	72%
F	AAC/AAG	54	26	48%
G	AAG/AAG	55	28	51%
H	AAG/ACG	71	36	51%
I	AGC/ACG	76	37	49%
L	AGT/AAC	67	30	45%

**Table 5:** Comparison of F_st_ values for each phenotype pair.

	DB	LB	VB	SS	RS
**DB**	0.00				
**LB**	0.01	0.00			
**VB**	0.00	0.00	0.00		
**SS**	0.01	0.00	0.00	0.00	
**RS**	0.23	0.16	0.26	0.28	0.00

F_st_ values between groups of individuals defined by the five categories of skin colour patter were computed on the basis of AFLP data by means of AFLP-SURV [50].

The related p-values were always <<0.00. DB diagonal bars; LB longitudinal bars; VB vertical bars; SS stellate spot; RS round spot.

**Figure 6:**
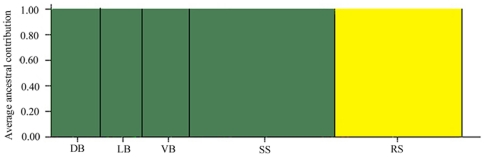
Illustration of the estimated population subdivision and the prediction of historical admixture. Two partitions (*K*) were empirically determined by STRUCTURE (version 2.3.3) for AFLP data aggregated by skin colour pattern, using a burn-in period of 100,000 steps, followed by 500,000 steps of data collection to ensure the convergence of the MCMC. Yellow bars refer to the northern genotype, here represented by round spot (RS) individuals. Green bars refer to the southern genotype, here represented by individuals showing the southern phenotypes stellate spot (SS), diagonal bars (DB), longitudinal bars (LB) and vertical bars (VB).

The estimation of the most likely number of groups, K, was two, which means that AFLPs were able to identify 2 distinct lineages, here called AFLP-A and AFLP-B. The bar plot re-arrangement based on skin colour pattern (Figure 6) suggested a unique relationship between round spot skin colour pattern and AFLP-B: the prediction of historical admixture within each population performed with two partitions indicated that all samples showing the round spot pattern were assigned to the AFLP-B group. In contrast, none of the round spot individuals were assigned to AFLP-A. Thus, AFLPs are completely linked to colour skin patterns. On the other hand, the spatial distribution of the two AFLP groups are not so clear. All the samples from continental Europe showed the same genotype, AFLP-B, with only one individual from the Danube river with genotype AFLP-A; populations from Italy had both AFLP-A and AFLP-B. AFLP-B genotype has been identified in 45 Italian individuals scattered in several, recently repopulated populations [pp, personal communication]. The Mantel test correlating differences in AFLP between populations and geographic distances did not show any significant correlation (R = 0.039, P = 0.68). Thus, whereas in continental Europe only AFLP-B genotypes are present, in Italy both AFLP genotypes can be found, even sympatric, and this prevents a clear geographic separation of the genotypes.

The Italian populations where both AFLP-A and AFLP-B are present are the ones with the highest values of polymorphism in AFLP: these had higher proportion of polymorphic loci at the 5% level and higher Hj values than the other populations. This scenario can be explained by the presence of stocked fish, increasing polymorphism. Only one population from continental Europe showed a great proportion of polymorphic loci (66.9) and a high Hj value (0.20), comparable to the Italian populations with both genotypes; this population is from the Danube river, the same one where also COI and Cytb provided evidence of previous contacts between the two groups of European pike.

### Identification of specific polymorphic AFLP fragments

**Figure 7:**
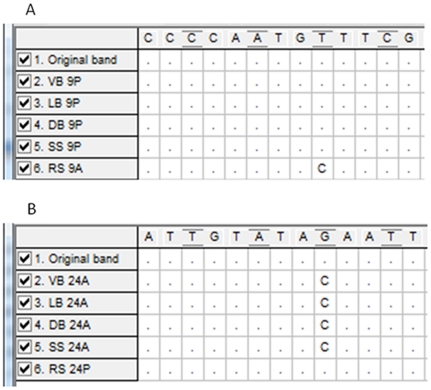
Sequences obtained for the specific polymorphic AFLP fragments. Sequences obtained for the band 9 (**A**) and the band 24 (**B**) for individuals showing the five phenotypes: stellate spot (SS), diagonal bars (DB), longitudinal bars (LB), vertical bars (VB) and round spot (RS). The original bands were obtained from a stellate spot individual for band 9 and from a round spot individual for band 24. 9P: band 9, present; 24P: band 24, present. 9A: band 9, absent; 24A: band 24, absent.

The sequences obtained for the two polymorphic bands (9, 24) did not show any similarity with other GenBank sequences; thus, we cannot suggest any specific location or function for them. A biunivocal relationship with skin colour pattern was found: all the individuals with round spot pattern showed the allele 24 whereas all the individuals with the other four skin patterns showed the allele 9 (Figure 7). Thus, these two highly informative nuclear SNPs represent an important and fast procedure for the rapid identification of the two groups of European pike.

### Conclusions

The northern pike is the most widespread Esocidae species in the world, and the only one naturally present in Europe, occupying a wide range of different lothic and lenthic habitats. It has the ability to tolerate very different prey typologies, salinity and temperatures, even though its life-cycle is strictly dependent on water vegetation. This plasticity was considered the biological factor supporting and facilitating the migration of individuals from the north to the south of the distribution area. Nevertheless, this research clearly demonstrated that behind such plasticity there might be the genetic, phenotypic and geographic distinction between two different taxonomic units.

The present analysis of the northern pike revealed a species complex, with one species widely distributed in Europe, Asia and North America and one in Italy only. The widely distributed species is more homogeneous in its skin colour patters, whereas the Italian one has a large variability in colour pattern. There is evidence of potential past connections between the two species, with individuals that have mismatches between their mtDNA, AFLP and phenotypic characters. Nevertheless, these individuals are present in areas where previous contact between the two species is plausible. The population in the Danube belongs to the widely distributed species, but some individuals have mtDNA loci of the Italian one, and even one individual with COI-A and Cytb-B. This may be a signature of introgression of the Italian species in northern areas in the past, when the paleo-delta of the Po River make the shores of the Adriatic sea closer to the present position of the Danube river [61]. On the contrary, most of the occurrences of the widely distributed species in Italy can be ascribed to recent introductions for recreational fishing. Such introductions potentially produced hybridisation between the two pike species, and this could explain the very few mismatches between phenotypes and genotypes. The large proportion of mismatches between phenotypes and genotypes in Maggiore Lake may suggest that, in this area, northern individuals could have migrated south in the past and maybe the history of hybridisation is here longer than the one due to recent human translocation of individuals.

In a previous evaluation of the stocking impact, a poor performance and a low level of introgression of stocked fries into a brackish northern pike population were reported [62]. In the case of Italian populations it is difficult to evaluate the real performance of stocked individuals, because no quantitative data on the repopulation with allochthonous specimens are available. This fact, together with the presence of allochthonous genotypes in several Italian populations here reported, suggests that the performance of stocked fish was sufficient to allow the persistence of allochtonous genotypes across generations. The findings here reported agree with the few introgressions registered for the Stege Nor population [62]. In fact, a strict association between the allochthonous genotype and the “new” round spot skin colour pattern is present in Italy, supporting the hypothesis that the stocked fish are at least in part reproductively separated by the autochthonous populations. This poor introgression is in contrast with data reported by Launey et al. [63] that found an extensive introgression between French populations and introduced stocks. The higher level of itnrogression in French populations compared to the Italian ones can be attributed to the fact that native French populations belong to the same widely distributed northern lineage used for introductions, whereas in Italy local populations belong to a different lineage, a separate species, for which hybridisation events are much rarer than sexual exchange between populations within the same species. French populations analysed by Launey et al. [42] in rivers with an outlet in the Mediterranean were all stocked, and not completely wild. Moreover, the autochthony of pike in Mediterranean France is dubious [54]. An alternative explanation for the different levels of introgression between the Italian and the French populations might be found in water temperature. The role of water temperature in pike biology is well established in differentiating reproductive periods [16] and French freshwaters temperatures may be more similar to those of other European countries than in Italian freshwater habitats, that are generally warmer.

The data reported here on statistically significant differences in mtDNA, AFLP, SNPs, skin colour pattern and number of scales in the lateral line support a clear differentiation between a northern lineage of pike, widely distributed in the palearctic region, and a southern lineage, distributed in Italy, and potentially in other areas presently or in the past connected to the Mediterranean (as the Danube area). All the analyses we performed show high concordance between genotypic and phenotypic markers to identify the two lineages. Moreover, a coalescent based approach on the tree topology for two different mtDNA loci and AFLP clustering supported the existence of the two independently evolving entities, that is, the genetic signature of two different species. The differentiation values between genetic markers, too high to belong to differentiation between populations, are still lower than those concerning the other Esocidae species; this suggests that the separation between the two species is relatively recent.

The type locality of *Esox lucius* is not specified in the original description, and it is only reported as “in Europa” [64]. We may assume that Linnaeus described the species from individuals from continental Europe, if not even from Sweden; thus, we suggest 1) to maintain the name *E. lucius* for the widespread northern lineage we identified in this analysis, and 2) to describe a new species for the southern samples showing the four skin colour pattern non present in continental Europe and having a lower number of scales in the lateral line. Surprisingly, no name has ever been proposed by taxonomists for the southern morphologies [65], thus, we propose the name of *Esox flaviae* n.sp. for the southern pike.

#### Species description

**SYSTEMATICS**

Phylum CHORDATA Bateson 1882

Subphylum VERTEBRATA Cuvier, 1812

Class ACTINOPTERYGII Klein, 1885

Subclass NEOPTERYGII Regan, 1923

Order ESOCIFORMES Nelson, 1994

Family Esocidae Crossman, 1996

Genus *Esox* Linnaeus, 1758

*Esox flaviae* sp. n.

(Figure 1A–D)

(urn:lsid:zoobank.org:act:A352D5B4-4EFC-44BB-A451-8D4F27CBB6FA)

#### Type locality

Lake Trasimeno (43° 8′ 43″N; 12° 5′ 52″E). It has a surface of 128 Km^2^, it is the fourth largest Italian lake and is localised in Central Italy in the Tevere river basin. It has been declared a Regional Italian Park (DLR 9/95) and includes two Natura 2000 sites (SIC IT5210018 and ZPS IT5210070). It is a closed laminar lake characterized by an extremely reduced depth (the mean depth is less than 5 m, with a maximum of 6.3 m). In this biotope a well-structured and well-studied population of the species is present [14], [16], [66], even if all the literature still refers to this population as to *E. lucius*.

#### Type material

Holotype: a male specimen in ethanol in a jar deposited at the Natural History Gallery of Casalina (http://www.unipg.it/camso1/galleria/g0.htm), Perugia, Italy, accession number GS1 Moreover, a 1.5 ml test tube containing the extracted DNA of this individual is present as accompanying material, with accession number GSDNA1.

Holotype male. Total Length 450 mm; Standard Length: 415 mm; Colour pattern: stellate spot.

#### Etymology

The specific name *flaviae* is the female genitive of Flavia, the name of the first women of the Flavian Dynasty, which included the emperor Titus Flavius (both his mother and his sister were named Flavia), to whom Pliny dedicated his *Naturalis Historia*. Four books of *Naturalis Historia* are devoted to zoology and to an attempt of systematics and they became a reference for subsequent naturalistic books. We dedicate the new species to the same person, to underline the “latin” origin of the species. Moreover, we propose the vernacular name of southern pike for the new species, to point out the different origin with respect to northern pike.

#### Differential diagnosis

As for northern pike, it is distinguishable from all other European freshwater fishes by large size, up to 1000 mm in total length; a long and flat duckbill-like snout; a large mouth with many sharp teeth on gill arches; rearward position of dorsal and anal fins with the dorsal one located far to the rear; pectoral and pelvic fins low on body, paired fins paddle-shaped [54].

It is possible to distinguish *E. flaviae* from the only congeneric species in Europe on the basis of several meristic characters (Table 6), which discriminate also between *E. flaviae* and all the other species of the genus. The strongest discrimination from *E. lucius sensu stricto* is possible on the basis of the number of scales in the lateral line: this number ranges from 101 to 115 in *E. flaviae* and from 125 to 148 in *E. lucius*. The two species differ also for the skin colour pattern. *E. flaviae* is very variable and shows four different colour pattern (stellate spot, diagonal bars, longitudinal bars and vertical bars, Fig. 1A–D), but never the colour pattern typical of *E. lucius* described as round spot (Fig. 1E).

**Table 6:** Meristic characters of esocids (Minimum-Maximum).

Name	Mandibular Pores	Pelvic rays	Pectoral rays	Scales in lateral line
***E. a. americanus***	4*	8–9*	14–15*	102–116*
***E. niger***	4*	9–10*	12–15*	117–135*
***E. masquinongy***	6–9*	11–12	14–19*	132–167*
***E. reichertii***	5*	-	-	130–163*
***E. lucius*** ** s.s.**	5*	9–10*	13–16*	125–148°
***E. flaviae***	3–9°	6–15°	4–19°	101–115°

*Raat, 1988;

° Our data.

#### Measurements (values referred to the population of the type locality)

Total length: 96–1000 mm (average 475 mm).

Standard length: 95% of total length circa.

Weight: average 0.8 kg [14] and up to above 10 kg.

Maximum age: 13 years

Length at first maturity: 300 mm

Dorsal spines (total): 1–2

Dorsal soft rays (total): 13–16

Anal spines: 1–2

Anal soft rays: 11–13

Caudal fin rays: 14–18

Mandibular pores: 3–8

Opercular pores: 3–8

#### Biology

As for the northern pike [54], the southern pike occurs in clear vegetated water bodies as lakes and large rivers. It is solitary and territorial, voracious predator feeding mainly on fishes, but also on frogs, crayfish and often cannibalistic. Males reproduce for the first time when one-year-old, females when two-years-old. As for northern pike, reproduction is closely related to the presence of submerged vegetation. Spawns in late winter (February-March) in central Italy and in early spring (March-April) in northern Italy. It is a valuable game fish for recreational fishing and may be impacted by habitat alteration and by competition and/or hybridisation with the northern pike previously used for stocking local populations in Italy.

#### Distribution

Central and northern Italy. Potentially, it can be present in other European water bodies in the Mediterranean area as those on the north-eastern shores of the Adriatic and in Mediterranean France. The individuals of *E. lucius* from the Danube river with genetic signature of introgression from *E. flaviae* have the typical phenotype of *E. lucius* (high number of scales, round spot skin colour pattern), and thus it is not likely that *E. flaviae* could be found in the Danube area.

#### DNA barcoding

GenBank accession number for holotype COI: HM563688.1 (COIhap1), holotype cytb: JN190460 (CytbIhap3).

GanBank accession numbers of additional individuals considered in this analyses for COI: HM563688- HM563707 (previously named COI-A), and for Cytb: JN190458 - JN190487 (previously named Cytb-A).

## Supporting Information

Text S1 **Distribution of** ***Esox lucius*** **.** In green: Countries where *Esox lucius* is considered native; in red: countries where is considered as introduced fish; yellow: countries for which incongruent informations are reported by different authors.
(DOC)

Text S2 **Samples origin.**
(DOC)

Text S3 **Original data for the 374 analysed samples.** N/A means data Not Available.
(DOC)

Text S4 **Permission to publish** **Figure 1** **–Monday, July 11^th^, 2011 17.08.**
(DOC)
