# Supplementary material for: Molecular and Phenotypic Evidence of a New Species of Genus Esox (Esocidae, Esociformes, Actinopterygii): The Southern Pike, Esox flaviae
Source: PLoS One. 2011 Dec 2;6(12):e25218. doi: 10.1371/journal.pone.0025218 (PMC3229480; doi:10.1371/journal.pone.0025218)
Supplement: Text S1 — Distribution of Esox lucius . In green: Countries where Esox lucius is considered native; in red: countries where is considered as introduced fish; yellow: countries for which incongruent informations are reported by different authors. (DOC) [file pone.0025218.s001.doc]

**SUPPLEMENTARY TEXT S1**

Distribution of *Esox lucius.* In green: Countries where *Esox lucius* is considered native; in red: countries where is considered as introduced fish; yellow: countries for which incongruent informations are reported by different authors


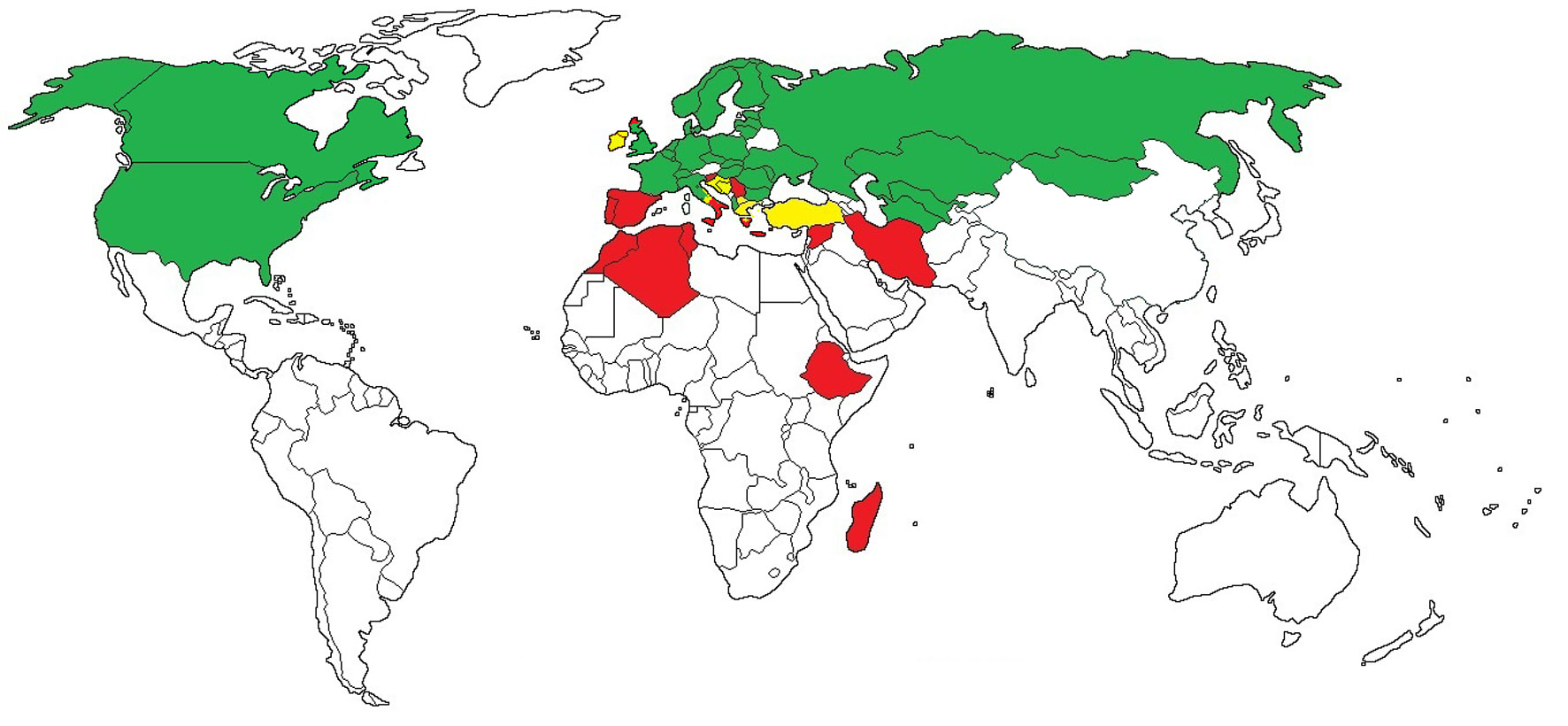


| **Country** | **ABB** | **Status** | **References (if known)** |
| --- | --- | --- | --- |
| Alaska | ALK | native |  |
| Albania | ALB | native |  |
| Algeria | DZA | introduced (from France) |  |
| Armenia | ARM | native |  |
| Azerbaijan | AZE | native |  |
| Azores Is | AZZ | introduced |  |
| Belgium | BEL | native |  |
| Bosnia Herzg | BIH | native |  |
| Bulgaria | BGR | native |  |
| Canada | CAN | native |  |
| China Main | CHN | native | Peter Manning,  .  .  (No Title). (.) |
| Croatia | HRV | native |  |
| Czech Rep | CZE | native |  |
| Denmark | DNK | native |  |
| Estonia | EST | native |  |
| Ethiopia | ETH | introduced |  |
| Finland | FIN | native |  |
| France | FRA | native |  |
| Georgia | GEO | native |  |
| Germany | DEU | native |  |
| Greece | GRC | native/introduced (European origin) | Economidis et al., 2000. Introduced and translocated fishes in the inland waters of Greece. Fisheries Management and Ecology, 7, 239-250. Welcomme, R.L. 1988 International introductions of inland aquatic species. FAO Fish. Tech. Pap. 294. 318 p. |
| Hungary | HUN | native |  |
| Iran |  |  | *Coad*, *B. W.* , 1996, Exotic and transplanted fishes in southwest Asia. *Publ*. *Espec*. *Inst*. *Esp*. *Oceanogr*., No. *21*, *81-106* |
| Ireland | IRL | introduced (from UK) | Elvira B., 2001. Identification of non-native freshwater fishes established in Europe and assessment of their potential threats to the biological diversity. CONVENTION ON THE CONSERVATION OF EUROPEAN WILDLIFE AND NATURAL HABITATS. Strasbourg, 11 December 2000; Raat, 1988 |
| Italy | ITA | native |  |
| Kazakhstan | KAZ | native |  |
| Latvia | LVA | native |  |
| Lithuania | LTU | native |  |
| Luxembourg | LUX | native |  |
| Macedonia | MKD | native |  |
| Madagascar | MDG | introduced (from France) | Raat, 1988 |
| Moldova Rep | MDA | native |  |
| Monaco | MCO | native |  |
| Mongolia | MNG | native |  |
| Morocco | MAR | introduced (from France) | Raat, 1988 |
| Netherlands | NLD | native |  |
| Norway | NOR | native |  |
| Poland | POL | native |  |
| Portugal | PRT | introduced (from unknow) | Holcík, J., 1991 Fish introductions in Europe with particular reference to its central and eastern part. Can. J. Fish. Aquat. Sci. 48 (Suppl. 1):13-23; Elvira B., 2001. Identification of non-native freshwater fishes established in Europe and assessment of their potential threats to the biological diversity. CONVENTION ON THE CONSERVATION OF EUROPEAN WILDLIFE AND NATURAL HABITATS. Strasbourg, 11 December 2000; Collares-Pereira et al., 2000. Threats imposed by water resources development schemes on the conservation of endangered fish species in the Guadiana River ; Raat, 1988 |
| Romania | ROM | native |  |
| Russian Fed | RUS | native |  |
| Serbia | SCG | native |  |
| Slovakia | SVK | native |  |
| Slovenia | SVN | native |  |
| Spain | ESP | introduced | Elvira B., 2001. Identification of non-native freshwater fishes established in Europe and assessment of their potential threats to the biological diversity. CONVENTION ON THE CONSERVATION OF EUROPEAN WILDLIFE AND NATURAL HABITATS. Strasbourg, 11 December 2000; Elvira B., 1998- Impact of introduced fish on the native freshwater fish fauna of Spain. In: "Stocking and Introduction of Fish", Cowx, I.G. (ed), Fishing News Books, Oxford, pp.: 186-190; Raat, 1988 |
| Sweden | SWE | native |  |
| Switzerland | CHE | native |  |
| Syria |  |  | *Coad*, *B. W.* , 1996, Exotic and transplanted fishes in southwest Asia. *Publ*. *Espec*. *Inst*. *Esp*. *Oceanogr*., No. *21*, *81-106* |
| Tunisia | TUN | introduced (from France) |  |
| Turkey | TUR | native/introduced | *Coad*, *B. W.* , 1996, Exotic and transplanted fishes in southwest Asia. *Publ*. *Espec*. *Inst*. *Esp*. *Oceanogr*., No. *21*, *81-106* |
| Turkmenistan | TKM | native |  |
| Uganda | UGA | introduced | Raat |
| UK | GBR | native |  |
| Ukraine | UKR | native |  |
| USA | USA | native |  |
| Uzbekistan | UZB | native |  |
