# Supplementary material for: Molecular and Phenotypic Evidence of a New Species of Genus Esox (Esocidae, Esociformes, Actinopterygii): The Southern Pike, Esox flaviae
Source: PLoS One. 2011 Dec 2;6(12):e25218. doi: 10.1371/journal.pone.0025218 (PMC3229480; doi:10.1371/journal.pone.0025218)
Supplement: Text S2 — Samples origin. (DOC) [file pone.0025218.s002.doc]

**SUPPLEMENTARY TEXT S2**

Samples origin.


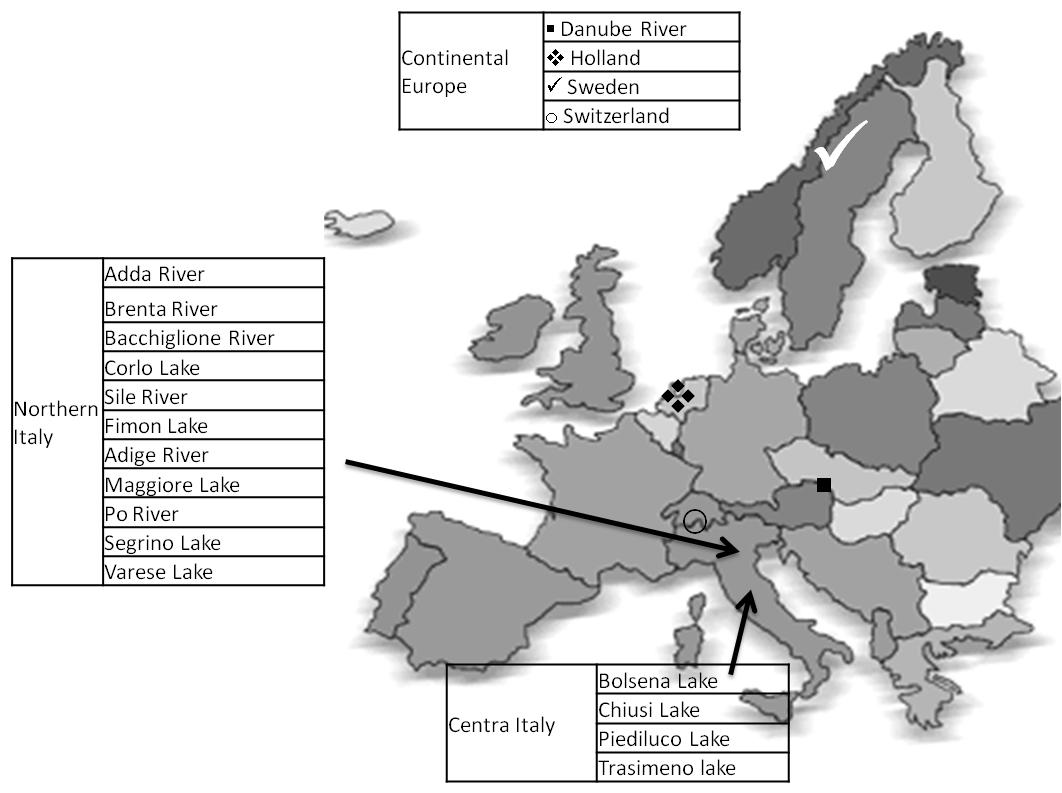


| **Sample ID** | **Origin** |
| --- | --- |
| 179 | Adda River |
| 180 | Adda River |
| 181 | Adda River |
| 182 | Adda River |
| 183 | Adda River |
| 184 | Adda River |
| 185 | Adda River |
| 186 | Adda River |
| 187 | Adda River |
| 188 | Adda River |
| 189 | Adda River |
| 190 | Adda River |
| 191 | Adda River |
| 192 | Adda River |
| 193 | Adda River |
| 194 | Adda River |
| 195 | Adda River |
| 196 | Adda River |
| 197 | Adda River |
| 198 | Adda River |
| 199 | Adda River |
| 200 | Adda River |
| 201 | Adda River |
| 202 | Adda River |
| 203 | Adda River |
| 204 | Adda River |
| 205 | Adda River |
| 720 | Adda River |
| 721 | Adda River |
| 722 | Adda River |
| 723 | Adda River |
| 724 | Adda River |
| 725 | Adda River |
| 726 | Adda River |
| 727 | Adda River |
| 728 | Adda River |
| 729 | Adda River |
| 730 | Adda River |
| 731 | Adda River |
| 732 | Adda River |
| 733 | Adda River |
| 734 | Adda River |
| 735 | Adda River |
| 736 | Adda River |
| 737 | Adda River |
| 1152 | Adige River |
| 1153 | Adige River |
| 1154 | Adige River |
| 1155 | Adige River |
| 1156 | Adige River |
| 1157 | Adige River |
| 1164 | Bacchiglione River |
| 1165 | Bacchiglione River |
| 1166 | Bacchiglione River |
| 1167 | Bacchiglione River |
| 1168 | Bacchiglione River |
| 1169 | Bacchiglione River |
| 1170 | Bacchiglione River |
| 1171 | Bacchiglione River |
| 1172 | Bacchiglione River |
| 1173 | Bacchiglione River |
| 1174 | Bacchiglione River |
| 1175 | Bacchiglione River |
| 1176 | Bacchiglione River |
| 1177 | Bacchiglione River |
| 1178 | Bacchiglione River |
| 1179 | Bacchiglione River |
| 1180 | Bacchiglione River |
| 1181 | Bacchiglione River |
| 1182 | Bacchiglione River |
| 157 | Bolsena Lake |
| 158 | Bolsena Lake |
| 159 | Bolsena Lake |
| 160 | Bolsena Lake |
| 161 | Bolsena Lake |
| 162 | Bolsena Lake |
| 163 | Bolsena Lake |
| 164 | Bolsena Lake |
| 165 | Bolsena Lake |
| 166 | Bolsena Lake |
| 167 | Bolsena Lake |
| 168 | Bolsena Lake |
| 169 | Bolsena Lake |
| 170 | Bolsena Lake |
| 171 | Bolsena Lake |
| 172 | Bolsena Lake |
| 173 | Bolsena Lake |
| 174 | Bolsena Lake |
| 175 | Bolsena Lake |
| 176 | Bolsena Lake |
| 177 | Bolsena Lake |
| 178 | Bolsena Lake |
| 1074 | Brenta River |
| 1075 | Brenta River |
| 1076 | Brenta River |
| 1077 | Brenta River |
| 1078 | Brenta River |
| 1079 | Brenta River |
| 1080 | Brenta River |
| 1081 | Brenta River |
| 1082 | Brenta River |
| 1083 | Brenta River |
| 1084 | Brenta River |
| 1085 | Brenta River |
| 1086 | Brenta River |
| 1087 | Brenta River |
| 1088 | Brenta River |
| 1278 | Brenta River |
| 1279 | Brenta River |
| 1280 | Brenta River |
| 1281 | Brenta River |
| 1282 | Brenta River |
| 1283 | Brenta River |
| 1284 | Brenta River |
| 1285 | Brenta River |
| 1286 | Brenta River |
| 1287 | Brenta River |
| 1288 | Brenta River |
| 1289 | Brenta River |
| 1290 | Brenta River |
| 1291 | Brenta River |
| 1292 | Brenta River |
| 1293 | Brenta River |
| 1294 | Brenta River |
| 1295 | Brenta River |
| 1296 | Brenta River |
| 1297 | Brenta River |
| 1298 | Brenta River |
| 1299 | Brenta River |
| 1300 | Brenta River |
| 1301 | Brenta River |
| 1302 | Brenta River |
| 1303 | Brenta River |
| 1304 | Brenta River |
| 1305 | Brenta River |
| 1306 | Brenta River |
| 63 | Chiusi Lake |
| 64 | Chiusi Lake |
| 65 | Chiusi Lake |
| 66 | Chiusi Lake |
| 67 | Chiusi Lake |
| 68 | Chiusi Lake |
| 69 | Chiusi Lake |
| 70 | Chiusi Lake |
| 71 | Chiusi Lake |
| 72 | Chiusi Lake |
| 73 | Chiusi Lake |
| 74 | Chiusi Lake |
| 75 | Chiusi Lake |
| 76 | Chiusi Lake |
| 77 | Chiusi Lake |
| 78 | Chiusi Lake |
| 79 | Chiusi Lake |
| 80 | Chiusi Lake |
| 81 | Chiusi Lake |
| 82 | Chiusi Lake |
| 83 | Chiusi Lake |
| 84 | Chiusi Lake |
| 85 | Chiusi Lake |
| 86 | Chiusi Lake |
| 87 | Chiusi Lake |
| 88 | Chiusi Lake |
| 89 | Chiusi Lake |
| 90 | Chiusi Lake |
| 260 | Chiusi Lake |
| 261 | Chiusi Lake |
| 262 | Chiusi Lake |
| 263 | Chiusi Lake |
| 264 | Chiusi Lake |
| 265 | Chiusi Lake |
| 266 | Chiusi Lake |
| 267 | Chiusi Lake |
| 268 | Chiusi Lake |
| 269 | Chiusi Lake |
| 270 | Chiusi Lake |
| 271 | Chiusi Lake |
| 272 | Chiusi Lake |
| 273 | Chiusi Lake |
| 274 | Chiusi Lake |
| 275 | Chiusi Lake |
| 276 | Chiusi Lake |
| 277 | Chiusi Lake |
| 278 | Chiusi Lake |
| 279 | Chiusi Lake |
| 280 | Chiusi Lake |
| 281 | Chiusi Lake |
| 282 | Chiusi Lake |
| 283 | Chiusi Lake |
| 284 | Chiusi Lake |
| 285 | Chiusi Lake |
| 286 | Chiusi Lake |
| 287 | Chiusi Lake |
| 288 | Chiusi Lake |
| 289 | Chiusi Lake |
| 290 | Chiusi Lake |
| 291 | Chiusi Lake |
| 292 | Chiusi Lake |
| 293 | Chiusi Lake |
| 294 | Chiusi Lake |
| 295 | Chiusi Lake |
| 296 | Chiusi Lake |
| 297 | Chiusi Lake |
| 298 | Chiusi Lake |
| 299 | Chiusi Lake |
| 300 | Chiusi Lake |
| 1158 | Corlo Lake |
| 1159 | Corlo Lake |
| 1160 | Corlo Lake |
| 1161 | Corlo Lake |
| 1162 | Corlo Lake |
| 1163 | Corlo Lake |
| 114 | Danube River |
| 115 | Danube River |
| 116 | Danube River |
| 117 | Danube River |
| 118 | Danube River |
| 119 | Danube River |
| 120 | Danube River |
| 121 | Danube River |
| 122 | Danube River |
| 123 | Danube River |
| 124 | Danube River |
| 125 | Danube River |
| 126 | Danube River |
| 127 | Danube River |
| 128 | Danube River |
| 129 | Danube River |
| 130 | Danube River |
| 131 | Danube River |
| 132 | Danube River |
| 133 | Danube River |
| 134 | Danube River |
| 135 | Danube River |
| 136 | Danube River |
| 206 | Danube River |
| 207 | Danube River |
| 208 | Danube River |
| 209 | Danube River |
| 210 | Danube River |
| 211 | Danube River |
| 212 | Danube River |
| 213 | Danube River |
| 214 | Danube River |
| 215 | Danube River |
| 216 | Danube River |
| 217 | Danube River |
| 218 | Danube River |
| 219 | Danube River |
| 220 | Danube River |
| 221 | Danube River |
| 222 | Danube River |
| 223 | Danube River |
| 224 | Danube River |
| 225 | Danube River |
| 226 | Danube River |
| 227 | Danube River |
| 228 | Danube River |
| 229 | Danube River |
| 1095 | Fimon Lake |
| 1096 | Fimon Lake |
| 1097 | Fimon Lake |
| 1098 | Fimon Lake |
| 1099 | Fimon Lake |
| 1100 | Fimon Lake |
| 1101 | Fimon Lake |
| 1102 | Fimon Lake |
| 1103 | Fimon Lake |
| 695 | Garlate Lake |
| 696 | Garlate Lake |
| 697 | Garlate Lake |
| 698 | Garlate Lake |
| 699 | Garlate Lake |
| 700 | Garlate Lake |
| 701 | Garlate Lake |
| 702 | Garlate Lake |
| 703 | Garlate Lake |
| 704 | Garlate Lake |
| 705 | Garlate Lake |
| 706 | Garlate Lake |
| 707 | Garlate Lake |
| 708 | Garlate Lake |
| 709 | Garlate Lake |
| 144 | Lago Maggiore |
| 145 | Lago Maggiore |
| 146 | Lago Maggiore |
| 147 | Lago Maggiore |
| 148 | Lago Maggiore |
| 149 | Lago Maggiore |
| 150 | Lago Maggiore |
| 151 | Lago Maggiore |
| 152 | Lago Maggiore |
| 153 | Lago Maggiore |
| 154 | Lago Maggiore |
| 155 | Lago Maggiore |
| 156 | Lago Maggiore |
| 689 | Montedoglio Lake |
| 690 | Montedoglio Lake |
| 691 | Montedoglio Lake |
| 692 | Montedoglio Lake |
| 693 | Montedoglio Lake |
| 694 | Montedoglio Lake |
| 710 | Nederland |
| 711 | Nederland |
| 712 | Nederland |
| **326** | Piediluco Lake |
| **327** | Piediluco Lake |
| 328 | Piediluco Lake |
| **329** | Piediluco Lake |
| 330 | Piediluco Lake |
| **331** | Piediluco Lake |
| 332 | Piediluco Lake |
| 343 | Piediluco Lake |
| 344 | Piediluco Lake |
| 345 | Piediluco Lake |
| 346 | Piediluco Lake |
| 347 | Piediluco Lake |
| 348 | Piediluco Lake |
| 349 | Piediluco Lake |
| 350 | Piediluco Lake |
| 351 | Piediluco Lake |
| 352 | Piediluco Lake |
| 353 | Piediluco Lake |
| 354 | Piediluco Lake |
| 355 | Piediluco Lake |
| 356 | Piediluco Lake |
| 357 | Piediluco Lake |
| 358 | Piediluco Lake |
| 359 | Piediluco Lake |
| 360 | Piediluco Lake |
| 361 | Piediluco Lake |
| 362 | Piediluco Lake |
| 94 | Po River |
| 95 | Po River |
| 96 | Po River |
| 97 | Po River |
| 98 | Po River |
| 99 | Po River |
| 100 | Po River |
| 101 | Po River |
| 102 | Po River |
| 103 | Po River |
| 104 | Po River |
| 105 | Po River |
| 106 | Po River |
| 107 | Po River |
| 108 | Po River |
| 109 | Po River |
| 110 | Po River |
| 111 | Po River |
| 112 | Po River |
| 113 | Po River |
| 137 | Po River |
| 138 | Po River |
| 139 | Po River |
| 140 | Po River |
| 141 | Po River |
| 142 | Po River |
| 143 | Po River |
| 1121 | Po River |
| 1122 | Po River |
| 1123 | Po River |
| 1124 | Po River |
| 1125 | Po River |
| 1126 | Po River |
| 1127 | Po River |
| 1128 | Po River |
| 1129 | Po River |
| 1130 | Po River |
| 1131 | Po River |
| 1132 | Po River |
| 1133 | Po River |
| 1134 | Po River |
| 1135 | Po River |
| 1136 | Po River |
| 1137 | Po River |
| 1138 | Po River |
| 1139 | Po River |
| 1140 | Po River |
| 1141 | Po River |
| 1142 | Po River |
| 1143 | Po River |
| 1144 | Po River |
| 1145 | Po River |
| 1146 | Po River |
| 1147 | Po River |
| 1148 | Po River |
| 1149 | Po River |
| 1150 | Po River |
| 1151 | Po River |
| 1261 | Po River |
| 1262 | Po River |
| 1263 | Po River |
| 1264 | Po River |
| 1265 | Po River |
| 1266 | Po River |
| 1267 | Po River |
| 1268 | Po River |
| 1269 | Po River |
| 1270 | Po River |
| 1271 | Po River |
| 1272 | Po River |
| 1273 | Po River |
| 1274 | Po River |
| 1275 | Po River |
| 1276 | Po River |
| 1277 | Po River |
| 1203 | Po River |
| 1204 | Po River |
| 1205 | Po River |
| 1206 | Po River |
| 1207 | Po River |
| 1208 | Po River |
| 1209 | Po River |
| 1210 | Po River |
| 363 | Segrino Lake |
| 364 | Segrino Lake |
| 365 | Segrino Lake |
| 366 | Segrino Lake |
| 367 | Segrino Lake |
| 368 | Segrino Lake |
| 369 | Segrino Lake |
| 370 | Segrino Lake |
| 371 | Segrino Lake |
| 372 | Segrino Lake |
| 373 | Segrino Lake |
| 1089 | Sile River |
| 1090 | Sile River |
| 1091 | Sile River |
| 1092 | Sile River |
| 1093 | Sile River |
| 1094 | Sile River |
| 1183 | Sile River |
| 1184 | Sile River |
| 1185 | Sile River |
| 1186 | Sile River |
| 1187 | Sile River |
| 1188 | Sile River |
| 1189 | Sile River |
| 1190 | Sile River |
| 1191 | Sile River |
| 1192 | Sile River |
| 1193 | Sile River |
| 1194 | Sile River |
| 1195 | Sile River |
| 1196 | Sile River |
| 1197 | Sile River |
| 1198 | Sile River |
| 1199 | Sile River |
| 1104 | Sweden |
| 1105 | Sweden |
| 1106 | Sweden |
| 1107 | Sweden |
| 1108 | Sweden |
| 1109 | Sweden |
| 1110 | Sweden |
| 1111 | Sweden |
| 1112 | Sweden |
| 23 | switzerland |
| 24 | switzerland |
| 25 | switzerland |
| 26 | switzerland |
| 713 | Torbiere Iseo |
| 714 | Torbiere Iseo |
| 715 | Torbiere Iseo |
| 716 | Torbiere Iseo |
| 717 | Torbiere Iseo |
| 718 | Torbiere Iseo |
| 719 | Torbiere Iseo |
| 759 | Trasimeno |
| 230 | Trasimeno Lake |
| 231 | Trasimeno Lake |
| 232 | Trasimeno Lake |
| 233 | Trasimeno Lake |
| 234 | Trasimeno Lake |
| 235 | Trasimeno Lake |
| 236 | Trasimeno Lake |
| 237 | Trasimeno Lake |
| 238 | Trasimeno Lake |
| 239 | Trasimeno Lake |
| 240 | Trasimeno Lake |
| 241 | Trasimeno Lake |
| 242 | Trasimeno Lake |
| 243 | Trasimeno Lake |
| 244 | Trasimeno Lake |
| 245 | Trasimeno Lake |
| 246 | Trasimeno Lake |
| 247 | Trasimeno Lake |
| 248 | Trasimeno Lake |
| 249 | Trasimeno Lake |
| 250 | Trasimeno Lake |
| 251 | Trasimeno Lake |
| 252 | Trasimeno Lake |
| 253 | Trasimeno Lake |
| 254 | Trasimeno Lake |
| 255 | Trasimeno Lake |
| 256 | Trasimeno Lake |
| 257 | Trasimeno Lake |
| 258 | Trasimeno Lake |
| 259 | Trasimeno Lake |
| 301 | Trasimeno Lake |
| 302 | Trasimeno Lake |
| 303 | Trasimeno Lake |
| 304 | Trasimeno Lake |
| 305 | Trasimeno Lake |
| 306 | Trasimeno Lake |
| 307 | Trasimeno Lake |
| 308 | Trasimeno Lake |
| 309 | Trasimeno Lake |
| 310 | Trasimeno Lake |
| 311 | Trasimeno Lake |
| 312 | Trasimeno Lake |
| 313 | Trasimeno Lake |
| 314 | Trasimeno Lake |
| 315 | Trasimeno Lake |
| 316 | Trasimeno Lake |
| 317 | Trasimeno Lake |
| 318 | Trasimeno Lake |
| 319 | Trasimeno Lake |
| 320 | Trasimeno Lake |
| 321 | Trasimeno Lake |
| 322 | Trasimeno Lake |
| 323 | Trasimeno Lake |
| 324 | Trasimeno Lake |
| 333 | Trasimeno Lake |
| 334 | Trasimeno Lake |
| 335 | Trasimeno Lake |
| 336 | Trasimeno Lake |
| 337 | Trasimeno Lake |
| 338 | Trasimeno Lake |
| 339 | Trasimeno Lake |
| 340 | Trasimeno Lake |
| 341 | Trasimeno Lake |
| 342 | Trasimeno Lake |
| 374 | Trasimeno Lake |
| 375 | Trasimeno Lake |
| 376 | Trasimeno Lake |
| 377 | Trasimeno Lake |
| 378 | Trasimeno Lake |
| 379 | Trasimeno Lake |
| 380 | Trasimeno Lake |
| 381 | Trasimeno Lake |
| 382 | Trasimeno Lake |
| 383 | Trasimeno Lake |
| 384 | Trasimeno Lake |
| 385 | Trasimeno Lake |
| 386 | Trasimeno Lake |
| 387 | Trasimeno Lake |
| 388 | Trasimeno Lake |
| 389 | Trasimeno Lake |
| 390 | Trasimeno Lake |
| 391 | Trasimeno Lake |
| 392 | Trasimeno Lake |
| 393 | Trasimeno Lake |
| 394 | Trasimeno Lake |
| 395 | Trasimeno Lake |
| 396 | Trasimeno Lake |
| 397 | Trasimeno Lake |
| 398 | Trasimeno Lake |
| 399 | Trasimeno Lake |
| 400 | Trasimeno Lake |
| 401 | Trasimeno Lake |
| 402 | Trasimeno Lake |
| 403 | Trasimeno Lake |
| 404 | Trasimeno Lake |
| 405 | Trasimeno Lake |
| 406 | Trasimeno Lake |
| 407 | Trasimeno Lake |
| 408 | Trasimeno Lake |
| 409 | Trasimeno Lake |
| 410 | Trasimeno Lake |
| 411 | Trasimeno Lake |
| 412 | Trasimeno Lake |
| 413 | Trasimeno Lake |
| 414 | Trasimeno Lake |
| 415 | Trasimeno Lake |
| 416 | Trasimeno Lake |
| 417 | Trasimeno Lake |
| 418 | Trasimeno Lake |
| 419 | Trasimeno Lake |
| 420 | Trasimeno Lake |
| 421 | Trasimeno Lake |
| 422 | Trasimeno Lake |
| 423 | Trasimeno Lake |
| 424 | Trasimeno Lake |
| 425 | Trasimeno Lake |
| 426 | Trasimeno Lake |
| 427 | Trasimeno Lake |
| 428 | Trasimeno Lake |
| 429 | Trasimeno Lake |
| 430 | Trasimeno Lake |
| 431 | Trasimeno Lake |
| 432 | Trasimeno Lake |
| 433 | Trasimeno Lake |
| 434 | Trasimeno Lake |
| 435 | Trasimeno Lake |
| 436 | Trasimeno Lake |
| 437 | Trasimeno Lake |
| 438 | Trasimeno Lake |
| 439 | Trasimeno Lake |
| 440 | Trasimeno Lake |
| 441 | Trasimeno Lake |
| 442 | Trasimeno Lake |
| 443 | Trasimeno Lake |
| 444 | Trasimeno Lake |
| 445 | Trasimeno Lake |
| 446 | Trasimeno Lake |
| 447 | Trasimeno Lake |
| 448 | Trasimeno Lake |
| 449 | Trasimeno Lake |
| 450 | Trasimeno Lake |
| 451 | Trasimeno Lake |
| 452 | Trasimeno Lake |
| 453 | Trasimeno Lake |
| 454 | Trasimeno Lake |
| 455 | Trasimeno Lake |
| 456 | Trasimeno Lake |
| 457 | Trasimeno Lake |
| 458 | Trasimeno Lake |
| 459 | Trasimeno Lake |
| 460 | Trasimeno Lake |
| 461 | Trasimeno Lake |
| 462 | Trasimeno Lake |
| 463 | Trasimeno Lake |
| 464 | Trasimeno Lake |
| 465 | Trasimeno Lake |
| 466 | Trasimeno Lake |
| 467 | Trasimeno Lake |
| 468 | Trasimeno Lake |
| 469 | Trasimeno Lake |
| 470 | Trasimeno Lake |
| 471 | Trasimeno Lake |
| 472 | Trasimeno Lake |
| 473 | Trasimeno Lake |
| 474 | Trasimeno Lake |
| 475 | Trasimeno Lake |
| 476 | Trasimeno Lake |
| 477 | Trasimeno Lake |
| 478 | Trasimeno Lake |
| 479 | Trasimeno Lake |
| 480 | Trasimeno Lake |
| 481 | Trasimeno Lake |
| 482 | Trasimeno Lake |
| 483 | Trasimeno Lake |
| 484 | Trasimeno Lake |
| 485 | Trasimeno Lake |
| 486 | Trasimeno Lake |
| 487 | Trasimeno Lake |
| 488 | Trasimeno Lake |
| 489 | Trasimeno Lake |
| 490 | Trasimeno Lake |
| 491 | Trasimeno Lake |
| 492 | Trasimeno Lake |
| 493 | Trasimeno Lake |
| 494 | Trasimeno Lake |
| 495 | Trasimeno Lake |
| 496 | Trasimeno Lake |
| 497 | Trasimeno Lake |
| 498 | Trasimeno Lake |
| 499 | Trasimeno Lake |
| 500 | Trasimeno Lake |
| 501 | Trasimeno Lake |
| 502 | Trasimeno Lake |
| 503 | Trasimeno Lake |
| 504 | Trasimeno Lake |
| 505 | Trasimeno Lake |
| 506 | Trasimeno Lake |
| 507 | Trasimeno Lake |
| 508 | Trasimeno Lake |
| 509 | Trasimeno Lake |
| 510 | Trasimeno Lake |
| 511 | Trasimeno Lake |
| 512 | Trasimeno Lake |
| 513 | Trasimeno Lake |
| 514 | Trasimeno Lake |
| 515 | Trasimeno Lake |
| 516 | Trasimeno Lake |
| 517 | Trasimeno Lake |
| 518 | Trasimeno Lake |
| 519 | Trasimeno Lake |
| 520 | Trasimeno Lake |
| 521 | Trasimeno Lake |
| 522 | Trasimeno Lake |
| 523 | Trasimeno Lake |
| 524 | Trasimeno Lake |
| 525 | Trasimeno Lake |
| 526 | Trasimeno Lake |
| 527 | Trasimeno Lake |
| 528 | Trasimeno Lake |
| 529 | Trasimeno Lake |
| 530 | Trasimeno Lake |
| 531 | Trasimeno Lake |
| 532 | Trasimeno Lake |
| 533 | Trasimeno Lake |
| 534 | Trasimeno Lake |
| 535 | Trasimeno Lake |
| 536 | Trasimeno Lake |
| 537 | Trasimeno Lake |
| 538 | Trasimeno Lake |
| 539 | Trasimeno Lake |
| 540 | Trasimeno Lake |
| 541 | Trasimeno Lake |
| 542 | Trasimeno Lake |
| 543 | Trasimeno Lake |
| 544 | Trasimeno Lake |
| 545 | Trasimeno Lake |
| 546 | Trasimeno Lake |
| 547 | Trasimeno Lake |
| 548 | Trasimeno Lake |
| 549 | Trasimeno Lake |
| 550 | Trasimeno Lake |
| 551 | Trasimeno Lake |
| 552 | Trasimeno Lake |
| 553 | Trasimeno Lake |
| 554 | Trasimeno Lake |
| 555 | Trasimeno Lake |
| 556 | Trasimeno Lake |
| 557 | Trasimeno Lake |
| 558 | Trasimeno Lake |
| 559 | Trasimeno Lake |
| 560 | Trasimeno Lake |
| 561 | Trasimeno Lake |
| 562 | Trasimeno Lake |
| 563 | Trasimeno Lake |
| 564 | Trasimeno Lake |
| 565 | Trasimeno Lake |
| 566 | Trasimeno Lake |
| 567 | Trasimeno Lake |
| 568 | Trasimeno Lake |
| 569 | Trasimeno Lake |
| 570 | Trasimeno Lake |
| 571 | Trasimeno Lake |
| 572 | Trasimeno Lake |
| 573 | Trasimeno Lake |
| 574 | Trasimeno Lake |
| 575 | Trasimeno Lake |
| 576 | Trasimeno Lake |
| 577 | Trasimeno Lake |
| 578 | Trasimeno Lake |
| 579 | Trasimeno Lake |
| 580 | Trasimeno Lake |
| 581 | Trasimeno Lake |
| 582 | Trasimeno Lake |
| 583 | Trasimeno Lake |
| 584 | Trasimeno Lake |
| 585 | Trasimeno Lake |
| 586 | Trasimeno Lake |
| 587 | Trasimeno Lake |
| 588 | Trasimeno Lake |
| 589 | Trasimeno Lake |
| 590 | Trasimeno Lake |
| 591 | Trasimeno Lake |
| 592 | Trasimeno Lake |
| 593 | Trasimeno Lake |
| 594 | Trasimeno Lake |
| 595 | Trasimeno Lake |
| 596 | Trasimeno Lake |
| 597 | Trasimeno Lake |
| 598 | Trasimeno Lake |
| 599 | Trasimeno Lake |
| 600 | Trasimeno Lake |
| 601 | Trasimeno Lake |
| 602 | Trasimeno Lake |
| 603 | Trasimeno Lake |
| 604 | Trasimeno Lake |
| 605 | Trasimeno Lake |
| 606 | Trasimeno Lake |
| 607 | Trasimeno Lake |
| 608 | Trasimeno Lake |
| 609 | Trasimeno Lake |
| 610 | Trasimeno Lake |
| 611 | Trasimeno Lake |
| 612 | Trasimeno Lake |
| 613 | Trasimeno Lake |
| 614 | Trasimeno Lake |
| 615 | Trasimeno Lake |
| 616 | Trasimeno Lake |
| 617 | Trasimeno Lake |
| 618 | Trasimeno Lake |
| 619 | Trasimeno Lake |
| 620 | Trasimeno Lake |
| 621 | Trasimeno Lake |
| 622 | Trasimeno Lake |
| 623 | Trasimeno Lake |
| 624 | Trasimeno Lake |
| 625 | Trasimeno Lake |
| 626 | Trasimeno Lake |
| 627 | Trasimeno Lake |
| 628 | Trasimeno Lake |
| 629 | Trasimeno Lake |
| 630 | Trasimeno Lake |
| 631 | Trasimeno Lake |
| 632 | Trasimeno Lake |
| 633 | Trasimeno Lake |
| 634 | Trasimeno Lake |
| 635 | Trasimeno Lake |
| 636 | Trasimeno Lake |
| 637 | Trasimeno Lake |
| 638 | Trasimeno Lake |
| 639 | Trasimeno Lake |
| 640 | Trasimeno Lake |
| 641 | Trasimeno Lake |
| 642 | Trasimeno Lake |
| 643 | Trasimeno Lake |
| 644 | Trasimeno Lake |
| 645 | Trasimeno Lake |
| 646 | Trasimeno Lake |
| 647 | Trasimeno Lake |
| 648 | Trasimeno Lake |
| 649 | Trasimeno Lake |
| 650 | Trasimeno Lake |
| 651 | Trasimeno Lake |
| 652 | Trasimeno Lake |
| 653 | Trasimeno Lake |
| 654 | Trasimeno Lake |
| 655 | Trasimeno Lake |
| 656 | Trasimeno Lake |
| 657 | Trasimeno Lake |
| 658 | Trasimeno Lake |
| 659 | Trasimeno Lake |
| 660 | Trasimeno Lake |
| 661 | Trasimeno Lake |
| 662 | Trasimeno Lake |
| 663 | Trasimeno Lake |
| 664 | Trasimeno Lake |
| 665 | Trasimeno Lake |
| 666 | Trasimeno Lake |
| 667 | Trasimeno Lake |
| 668 | Trasimeno Lake |
| 669 | Trasimeno Lake |
| 670 | Trasimeno Lake |
| 671 | Trasimeno Lake |
| 672 | Trasimeno Lake |
| 673 | Trasimeno Lake |
| 674 | Trasimeno Lake |
| 675 | Trasimeno Lake |
| 676 | Trasimeno Lake |
| 677 | Trasimeno Lake |
| 678 | Trasimeno Lake |
| 679 | Trasimeno Lake |
| 680 | Trasimeno Lake |
| 681 | Trasimeno Lake |
| 682 | Trasimeno Lake |
| 683 | Trasimeno Lake |
| 684 | Trasimeno Lake |
| 685 | Trasimeno Lake |
| 686 | Trasimeno Lake |
| 687 | Trasimeno Lake |
| 688 | Trasimeno Lake |
| 738 | Trasimeno Lake |
| 739 | Trasimeno Lake |
| 740 | Trasimeno Lake |
| 741 | Trasimeno Lake |
| 742 | Trasimeno Lake |
| 743 | Trasimeno Lake |
| 744 | Trasimeno Lake |
| 745 | Trasimeno Lake |
| 746 | Trasimeno Lake |
| 747 | Trasimeno Lake |
| 748 | Trasimeno Lake |
| 749 | Trasimeno Lake |
| 750 | Trasimeno Lake |
| 751 | Trasimeno Lake |
| 752 | Trasimeno Lake |
| 753 | Trasimeno Lake |
| 754 | Trasimeno Lake |
| 755 | Trasimeno Lake |
| 756 | Trasimeno Lake |
| 757 | Trasimeno Lake |
| 758 | Trasimeno Lake |
| 760 | Trasimeno Lake |
| 761 | Trasimeno Lake |
| 762 | Trasimeno Lake |
| 763 | Trasimeno Lake |
| 764 | Trasimeno Lake |
| 765 | Trasimeno Lake |
| 766 | Trasimeno Lake |
| 767 | Trasimeno Lake |
| 768 | Trasimeno Lake |
| 769 | Trasimeno Lake |
| 770 | Trasimeno Lake |
| 771 | Trasimeno Lake |
| 772 | Trasimeno Lake |
| 773 | Trasimeno Lake |
| 774 | Trasimeno Lake |
| 775 | Trasimeno Lake |
| 776 | Trasimeno Lake |
| 777 | Trasimeno Lake |
| 778 | Trasimeno Lake |
| 779 | Trasimeno Lake |
| 780 | Trasimeno Lake |
| 781 | Trasimeno Lake |
| 782 | Trasimeno Lake |
| 783 | Trasimeno Lake |
| 784 | Trasimeno Lake |
| 785 | Trasimeno Lake |
| 786 | Trasimeno Lake |
| 787 | Trasimeno Lake |
| 788 | Trasimeno Lake |
| 789 | Trasimeno Lake |
| 790 | Trasimeno Lake |
| 791 | Trasimeno Lake |
| 792 | Trasimeno Lake |
| 793 | Trasimeno Lake |
| 794 | Trasimeno Lake |
| 795 | Trasimeno Lake |
| 796 | Trasimeno Lake |
| 797 | Trasimeno Lake |
| 798 | Trasimeno Lake |
| 799 | Trasimeno Lake |
| 800 | Trasimeno Lake |
| 801 | Trasimeno Lake |
| 802 | Trasimeno Lake |
| 803 | Trasimeno Lake |
| 804 | Trasimeno Lake |
| 805 | Trasimeno Lake |
| 806 | Trasimeno Lake |
| 807 | Trasimeno Lake |
| 808 | Trasimeno Lake |
| 809 | Trasimeno Lake |
| 810 | Trasimeno Lake |
| 811 | Trasimeno Lake |
| 812 | Trasimeno Lake |
| 813 | Trasimeno Lake |
| 814 | Trasimeno Lake |
| 815 | Trasimeno Lake |
| 816 | Trasimeno Lake |
| 817 | Trasimeno Lake |
| 818 | Trasimeno Lake |
| 819 | Trasimeno Lake |
| 820 | Trasimeno Lake |
| 821 | Trasimeno Lake |
| 822 | Trasimeno Lake |
| 823 | Trasimeno Lake |
| 824 | Trasimeno Lake |
| 825 | Trasimeno Lake |
| 826 | Trasimeno Lake |
| 827 | Trasimeno Lake |
| 828 | Trasimeno Lake |
| 829 | Trasimeno Lake |
| 830 | Trasimeno Lake |
| 831 | Trasimeno Lake |
| 832 | Trasimeno Lake |
| 833 | Trasimeno Lake |
| 834 | Trasimeno Lake |
| 835 | Trasimeno Lake |
| 836 | Trasimeno Lake |
| 837 | Trasimeno Lake |
| 838 | Trasimeno Lake |
| 839 | Trasimeno Lake |
| 840 | Trasimeno Lake |
| 841 | Trasimeno Lake |
| 842 | Trasimeno Lake |
| 843 | Trasimeno Lake |
| 844 | Trasimeno Lake |
| 845 | Trasimeno Lake |
| 846 | Trasimeno Lake |
| 847 | Trasimeno Lake |
| 848 | Trasimeno Lake |
| 849 | Trasimeno Lake |
| 850 | Trasimeno Lake |
| 851 | Trasimeno Lake |
| 852 | Trasimeno Lake |
| 853 | Trasimeno Lake |
| 854 | Trasimeno Lake |
| 855 | Trasimeno Lake |
| 856 | Trasimeno Lake |
| 857 | Trasimeno Lake |
| 858 | Trasimeno Lake |
| 859 | Trasimeno Lake |
| 860 | Trasimeno Lake |
| 861 | Trasimeno Lake |
| 862 | Trasimeno Lake |
| 863 | Trasimeno Lake |
| 864 | Trasimeno Lake |
| 865 | Trasimeno Lake |
| 866 | Trasimeno Lake |
| 867 | Trasimeno Lake |
| 868 | Trasimeno Lake |
| 869 | Trasimeno Lake |
| 870 | Trasimeno Lake |
| 871 | Trasimeno Lake |
| 872 | Trasimeno Lake |
| 873 | Trasimeno Lake |
| 874 | Trasimeno Lake |
| 875 | Trasimeno Lake |
| 876 | Trasimeno Lake |
| 877 | Trasimeno Lake |
| 878 | Trasimeno Lake |
| 879 | Trasimeno Lake |
| 880 | Trasimeno Lake |
| 881 | Trasimeno Lake |
| 882 | Trasimeno Lake |
| 883 | Trasimeno Lake |
| 884 | Trasimeno Lake |
| 885 | Trasimeno Lake |
| 886 | Trasimeno Lake |
| 887 | Trasimeno Lake |
| 888 | Trasimeno Lake |
| 889 | Trasimeno Lake |
| 890 | Trasimeno Lake |
| 891 | Trasimeno Lake |
| 892 | Trasimeno Lake |
| 893 | Trasimeno Lake |
| 894 | Trasimeno Lake |
| 895 | Trasimeno Lake |
| 896 | Trasimeno Lake |
| 897 | Trasimeno Lake |
| 898 | Trasimeno Lake |
| 899 | Trasimeno Lake |
| 900 | Trasimeno Lake |
| 901 | Trasimeno Lake |
| 902 | Trasimeno Lake |
| 903 | Trasimeno Lake |
| 904 | Trasimeno Lake |
| 905 | Trasimeno Lake |
| 906 | Trasimeno Lake |
| 907 | Trasimeno Lake |
| 908 | Trasimeno Lake |
| 909 | Trasimeno Lake |
| 910 | Trasimeno Lake |
| 911 | Trasimeno Lake |
| 912 | Trasimeno Lake |
| 913 | Trasimeno Lake |
| 914 | Trasimeno Lake |
| 915 | Trasimeno Lake |
| 916 | Trasimeno Lake |
| 917 | Trasimeno Lake |
| 918 | Trasimeno Lake |
| 919 | Trasimeno Lake |
| 920 | Trasimeno Lake |
| 921 | Trasimeno Lake |
| 922 | Trasimeno Lake |
| 923 | Trasimeno Lake |
| 924 | Trasimeno Lake |
| 925 | Trasimeno Lake |
| 926 | Trasimeno Lake |
| 927 | Trasimeno Lake |
| 928 | Trasimeno Lake |
| 929 | Trasimeno Lake |
| 930 | Trasimeno Lake |
| 931 | Trasimeno Lake |
| 932 | Trasimeno Lake |
| 933 | Trasimeno Lake |
| 934 | Trasimeno Lake |
| 935 | Trasimeno Lake |
| 936 | Trasimeno Lake |
| 937 | Trasimeno Lake |
| 938 | Trasimeno Lake |
| 939 | Trasimeno Lake |
| 940 | Trasimeno Lake |
| 941 | Trasimeno Lake |
| 942 | Trasimeno Lake |
| 943 | Trasimeno Lake |
| 944 | Trasimeno Lake |
| 945 | Trasimeno Lake |
| 946 | Trasimeno Lake |
| 947 | Trasimeno Lake |
| 948 | Trasimeno Lake |
| 949 | Trasimeno Lake |
| 950 | Trasimeno Lake |
| 951 | Trasimeno Lake |
| 952 | Trasimeno Lake |
| 953 | Trasimeno Lake |
| 954 | Trasimeno Lake |
| 955 | Trasimeno Lake |
| 956 | Trasimeno Lake |
| 957 | Trasimeno Lake |
| 958 | Trasimeno Lake |
| 959 | Trasimeno Lake |
| 960 | Trasimeno Lake |
| 961 | Trasimeno Lake |
| 962 | Trasimeno Lake |
| 963 | Trasimeno Lake |
| 964 | Trasimeno Lake |
| 965 | Trasimeno Lake |
| 966 | Trasimeno Lake |
| 967 | Trasimeno Lake |
| 968 | Trasimeno Lake |
| 969 | Trasimeno Lake |
| 970 | Trasimeno Lake |
| 971 | Trasimeno Lake |
| 972 | Trasimeno Lake |
| 973 | Trasimeno Lake |
| 974 | Trasimeno Lake |
| 975 | Trasimeno Lake |
| 976 | Trasimeno Lake |
| 977 | Trasimeno Lake |
| 978 | Trasimeno Lake |
| 979 | Trasimeno Lake |
| 980 | Trasimeno Lake |
| 981 | Trasimeno Lake |
| 982 | Trasimeno Lake |
| 983 | Trasimeno Lake |
| 984 | Trasimeno Lake |
| 985 | Trasimeno Lake |
| 986 | Trasimeno Lake |
| 987 | Trasimeno Lake |
| 988 | Trasimeno Lake |
| 989 | Trasimeno Lake |
| 990 | Trasimeno Lake |
| 991 | Trasimeno Lake |
| 992 | Trasimeno Lake |
| 993 | Trasimeno Lake |
| 994 | Trasimeno Lake |
| 995 | Trasimeno Lake |
| 996 | Trasimeno Lake |
| 997 | Trasimeno Lake |
| 998 | Trasimeno Lake |
| 999 | Trasimeno Lake |
| 1000 | Trasimeno Lake |
| 1001 | Trasimeno Lake |
| 1002 | Trasimeno Lake |
| 1003 | Trasimeno Lake |
| 1004 | Trasimeno Lake |
| 1005 | Trasimeno Lake |
| 1006 | Trasimeno Lake |
| 1007 | Trasimeno Lake |
| 1008 | Trasimeno Lake |
| 1009 | Trasimeno Lake |
| 1010 | Trasimeno Lake |
| 1011 | Trasimeno Lake |
| 1012 | Trasimeno Lake |
| 1013 | Trasimeno Lake |
| 1014 | Trasimeno Lake |
| 1015 | Trasimeno Lake |
| 1016 | Trasimeno Lake |
| 1017 | Trasimeno Lake |
| 1018 | Trasimeno Lake |
| 1019 | Trasimeno Lake |
| 1020 | Trasimeno Lake |
| 1021 | Trasimeno Lake |
| 1022 | Trasimeno Lake |
| 1023 | Trasimeno Lake |
| 1024 | Trasimeno Lake |
| 1025 | Trasimeno Lake |
| 1026 | Trasimeno Lake |
| 1027 | Trasimeno Lake |
| 1028 | Trasimeno Lake |
| 1029 | Trasimeno Lake |
| 1030 | Trasimeno Lake |
| 1031 | Trasimeno Lake |
| 1032 | Trasimeno Lake |
| 1033 | Trasimeno Lake |
| 1034 | Trasimeno Lake |
| 1035 | Trasimeno Lake |
| 1036 | Trasimeno Lake |
| 1037 | Trasimeno Lake |
| 1038 | Trasimeno Lake |
| 1039 | Trasimeno Lake |
| 1040 | Trasimeno Lake |
| 1041 | Trasimeno Lake |
| 1042 | Trasimeno Lake |
| 1043 | Trasimeno Lake |
| 1044 | Trasimeno Lake |
| 1045 | Trasimeno Lake |
| 1046 | Trasimeno Lake |
| 1047 | Trasimeno Lake |
| 1048 | Trasimeno Lake |
| 1049 | Trasimeno Lake |
| 1050 | Trasimeno Lake |
| 1051 | Trasimeno Lake |
| 1052 | Trasimeno Lake |
| 1053 | Trasimeno Lake |
| 1054 | Trasimeno Lake |
| 1055 | Trasimeno Lake |
| 1056 | Trasimeno Lake |
| 1057 | Trasimeno Lake |
| 1058 | Trasimeno Lake |
| 1059 | Trasimeno Lake |
| 1060 | Trasimeno Lake |
| 1061 | Trasimeno Lake |
| 1062 | Trasimeno Lake |
| 1063 | Trasimeno Lake |
| 1064 | Trasimeno Lake |
| 1065 | Trasimeno Lake |
| 1066 | Trasimeno Lake |
| 1067 | Trasimeno Lake |
| 1068 | Trasimeno Lake |
| 1069 | Trasimeno Lake |
| 1070 | Trasimeno Lake |
| 1071 | Trasimeno Lake |
| 1072 | Trasimeno Lake |
| 1073 | Trasimeno Lake |
| 1211 | Trasimeno Lake |
| 1212 | Trasimeno Lake |
| 1213 | Trasimeno Lake |
| 1214 | Trasimeno Lake |
| 1215 | Trasimeno Lake |
| 1216 | Trasimeno Lake |
| 1217 | Trasimeno Lake |
| 1218 | Trasimeno Lake |
| 1219 | Trasimeno Lake |
| 1220 | Trasimeno Lake |
| 1221 | Trasimeno Lake |
| 1222 | Trasimeno Lake |
| 1223 | Trasimeno Lake |
| 1224 | Trasimeno Lake |
| 1225 | Trasimeno Lake |
| 1226 | Trasimeno Lake |
| 1227 | Trasimeno Lake |
| 1228 | Trasimeno Lake |
| 1229 | Trasimeno Lake |
| 1230 | Trasimeno Lake |
| 1231 | Trasimeno Lake |
| 1232 | Trasimeno Lake |
| 1233 | Trasimeno Lake |
| 1234 | Trasimeno Lake |
| 1235 | Trasimeno Lake |
| 1236 | Trasimeno Lake |
| 1237 | Trasimeno Lake |
| 1238 | Trasimeno Lake |
| 1239 | Trasimeno Lake |
| 1240 | Trasimeno Lake |
| 1241 | Trasimeno Lake |
| 1242 | Trasimeno Lake |
| 1243 | Trasimeno Lake |
| 1244 | Trasimeno Lake |
| 1245 | Trasimeno Lake |
| 1246 | Trasimeno Lake |
| 1247 | Trasimeno Lake |
| 1248 | Trasimeno Lake |
| 1249 | Trasimeno Lake |
| 1250 | Trasimeno Lake |
| 1251 | Trasimeno Lake |
| 1252 | Trasimeno Lake |
| 1253 | Trasimeno Lake |
| 1254 | Trasimeno Lake |
| 1255 | Trasimeno Lake |
| 1256 | Trasimeno Lake |
| 1257 | Trasimeno Lake |
| 1258 | Trasimeno Lake |
| 1259 | Trasimeno Lake |
| 1260 | Trasimeno Lake |
| 1307 | Trasimeno Lake |
| 1308 | Trasimeno Lake |
| 1309 | Trasimeno Lake |
| 1310 | Trasimeno Lake |
| 1311 | Trasimeno Lake |
| 1312 | Trasimeno Lake |
| 1313 | Trasimeno Lake |
| 1314 | Trasimeno Lake |
| 1315 | Trasimeno Lake |
| 1316 | Trasimeno Lake |
| 1317 | Trasimeno Lake |
| 1318 | Trasimeno Lake |
| 1319 | Trasimeno Lake |
| 1320 | Trasimeno Lake |
| 1321 | Trasimeno Lake |
| 1322 | Trasimeno Lake |
| 1323 | Trasimeno Lake |
| 1324 | Trasimeno Lake |
| 1 | Trasimeno Lake |
| 2 | Trasimeno Lake |
| 3 | Trasimeno Lake |
| 4 | Trasimeno Lake |
| 5 | Trasimeno Lake |
| 6 | Trasimeno Lake |
| 7 | Trasimeno Lake |
| 8 | Trasimeno Lake |
| 9 | Trasimeno Lake |
| 10 | Trasimeno Lake |
| 11 | Trasimeno Lake |
| 12 | Trasimeno Lake |
| 13 | Trasimeno Lake |
| 14 | Trasimeno Lake |
| 15 | Trasimeno Lake |
| 16 | Trasimeno Lake |
| 17 | Trasimeno Lake |
| 18 | Trasimeno Lake |
| 19 | Trasimeno Lake |
| 20 | Trasimeno Lake |
| 21 | Trasimeno Lake |
| 22 | Trasimeno Lake |
| 42 | Trasimeno Lake |
| 43 | Trasimeno Lake |
| 44 | Trasimeno Lake |
| 45 | Trasimeno Lake |
| 46 | Trasimeno Lake |
| 47 | Trasimeno Lake |
| 48 | Trasimeno Lake |
| 49 | Trasimeno Lake |
| 50 | Trasimeno Lake |
| 51 | Trasimeno Lake |
| 52 | Trasimeno Lake |
| 53 | Trasimeno Lake |
| 54 | Trasimeno Lake |
| 55 | Trasimeno Lake |
| 56 | Trasimeno Lake |
| 57 | Trasimeno Lake |
| 58 | Trasimeno Lake |
| 59 | Trasimeno Lake |
| 60 | Trasimeno Lake |
| 61 | Trasimeno Lake |
| 62 | Trasimeno Lake |
| 91 | Trasimeno Lake |
| 92 | Trasimeno Lake |
| 93 | Trasimeno Lake |
| 1113 | Varese Lake |
| 1114 | Varese Lake |
| 1115 | Varese Lake |
| 1116 | Varese Lake |
| 1117 | Varese Lake |
| 1118 | Varese Lake |
| 1119 | Varese Lake |
| 1120 | Varese Lake |
|  |  |
|  |  |
|  |  |
|  |  |
|  |  |
|  |  |
|  |  |
|  |  |
