# Supplementary material for: Molecular and Phenotypic Evidence of a New Species of Genus Esox (Esocidae, Esociformes, Actinopterygii): The Southern Pike, Esox flaviae
Source: PLoS One. 2011 Dec 2;6(12):e25218. doi: 10.1371/journal.pone.0025218 (PMC3229480; doi:10.1371/journal.pone.0025218)
Supplement: Text S3 — Original data for the 374 analysed samples. N/A means data Not Available. (DOC) [file pone.0025218.s003.doc]

**SUPPLEMENTARY TEXT S3**

Original data for the 374 analysed samples. N/A means data Not Available.

| Order | Origin | CytB haplogroup | CytB group | COI haplogroup | COI group | Lateral line scales | skin colour pattern |
| --- | --- | --- | --- | --- | --- | --- | --- |
| 123 | Brenta_River | CytBhap18 | B | COIhap12 | B | N/A | RS |
| 124 | Brenta_River | CytBhap17 | B | COIhap12 | B | N/A | RS |
| 125 | Brenta_River | CytBhap2 | B | COIhap12 | B | N/A | RS |
| 126 | Brenta_River | N/A | N/A | N/A | N/A | N/A | N/A |
| 127 | Brenta_River | N/A | N/A | N/A | N/A | N/A | N/A |
| 128 | Brenta_River | N/A | N/A | N/A | N/A | N/A | N/A |
| 129 | Brenta_River | N/A | N/A | N/A | N/A | N/A | N/A |
| 130 | Brenta_River | N/A | N/A | N/A | N/A | N/A | N/A |
| 131 | Brenta_River | N/A | N/A | N/A | N/A | N/A | N/A |
| 132 | Brenta_River | N/A | N/A | N/A | N/A | N/A | N/A |
| 133 | Brenta_River | N/A | N/A | N/A | N/A | N/A | N/A |
| 134 | Brenta_River | N/A | N/A | N/A | N/A | N/A | N/A |
| 135 | Brenta_River | N/A | N/A | N/A | N/A | N/A | N/A |
| 136 | Brenta_River | N/A | N/A | N/A | N/A | N/A | N/A |
| 137 | Brenta_River | N/A | N/A | N/A | N/A | N/A | N/A |
| 138 | Brenta_River | N/A | N/A | N/A | N/A | N/A | N/A |
| 139 | Brenta_River | N/A | N/A | N/A | N/A | N/A | N/A |
| 140 | Brenta_River | N/A | N/A | N/A | N/A | N/A | N/A |
| 141 | Brenta_River | N/A | N/A | N/A | N/A | N/A | N/A |
| 142 | Brenta_River | N/A | N/A | N/A | N/A | N/A | N/A |
| 143 | Brenta_River | N/A | N/A | N/A | N/A | N/A | N/A |
| 144 | Brenta_River | N/A | N/A | N/A | N/A | N/A | N/A |
| 145 | Brenta_River | CytBhap1 | B | COIhap12 | B | N/A | N/A |
| 146 | Brenta_River | N/A | N/A | N/A | N/A | N/A | N/A |
| 147 | Brenta_River | CytBhap1 | B | COIhap12 | B | N/A | N/A |
| 148 | Brenta_River | CytBhap2 | B | COIhap12 | B | N/A | N/A |
| 149 | Brenta_River | N/A | N/A | N/A | N/A | N/A | N/A |
| 150 | Brenta_River | N/A | N/A | N/A | N/A | N/A | N/A |
| 151 | Brenta_River | N/A | N/A | N/A | N/A | N/A | N/A |
| 9 | Trasimeno_Lake | CytBhap3 | A | COIhap1 | A | 103 | VB |
| 71 | Trasimeno_Lake | CytBhap3 | A | COIhap1 | A | 111 | SS |
| 56 | Trasimeno_Lake | CytBhap3 | A | COIhap1 | A | 110 | SS |
| 41 | Trasimeno_Lake | CytBhap3 | A | COIhap1 | A | 108 | SS |
| 42 | Trasimeno_Lake | N/A | N/A | COIhap1 | A | 108 | SS |
| 30 | Trasimeno_Lake | CytBhap3 | A | N/A | N/A | 106 | VB |
| 26 | Trasimeno_Lake | CytBhap3 | A | N/A | N/A | 105 | SS |
| 10 | Trasimeno_Lake | CytBhap3 | A | COIhap1 | A | 103 | VB |
| 72 | Trasimeno_Lake | N/A | N/A | COIhap1 | A | 111 | LB |
| 57 | Trasimeno_Lake | N/A | N/A | COIhap1 | A | 110 | VB |
| 52 | Trasimeno_Lake | N/A | N/A | COIhap1 | A | 109 | VB |
| 43 | Trasimeno_Lake | CytBhap3 | A | COIhap1 | A | 108 | DB |
| 31 | Trasimeno_Lake | N/A | N/A | COIhap1 | A | 106 | VB |
| 11 | Trasimeno_Lake | N/A | N/A | COIhap1 | A | 103 | DB |
| 58 | Trasimeno_Lake | CytBhap3 | A | COIhap1 | A | 110 | DB |
| 59 | Trasimeno_Lake | CytBhap3 | A | COIhap1 | A | 110 | SS |
| 20 | Chiusi _Lake | N/A | N/A | COIhap1 | A | 104 | SS |
| 152 | Chiusi _Lake | CytBhap3 | A | COIhap1 | A | N/A | RS |
| 32 | Chiusi _Lake | CytBhap4 | B | COIhap2 | B | 106 | LB |
| 38 | Chiusi _Lake | CytBhap3 | A | COIhap1 | A | 107 | LB |
| 153 | Chiusi _Lake | CytBhap4 | B | COIhap2 | B | N/A | RS |
| 60 | Chiusi _Lake | CytBhap3 | A | COIhap1 | A | 110 | LB |
| 154 | Chiusi _Lake | N/A | N/A | COIhap1 | A | N/A | RS |
| 155 | Chiusi _Lake | CytBhap5 | B | COIhap3 | B | N/A | RS |
| 156 | Chiusi _Lake | N/A | N/A | COIhap1 | A | N/A | LB |
| 157 | Chiusi _Lake | CytBhap4 | B | COIhap2 | B | N/A | RS |
| 158 | Chiusi _Lake | CytBhap3 | A | COIhap1 | A | N/A | LB |
| 159 | Chiusi _Lake | CytBhap3 | A | COIhap1 | A | N/A | RS |
| 44 | Chiusi _Lake | N/A | N/A | COIhap1 | A | 108 | SS |
| 33 | Chiusi _Lake | CytBhap3 | A | COIhap1 | A | 106 | LB |
| 12 | Chiusi _Lake | N/A | N/A | COIhap4 | A | 103 | SS |
| 39 | Chiusi _Lake | N/A | N/A | COIhap1 | A | 107 | SS |
| 80 | Chiusi _Lake | CytBhap3 | A | COIhap1 | A | 114 | SS |
| 81 | Chiusi _Lake | CytBhap3 | A | COIhap1 | A | 115 | SS |
| 160 | Chiusi _Lake | N/A | N/A | COIhap3 | B | N/A | RS |
| 161 | Chiusi _Lake | CytBhap3 | A | COIhap1 | A | N/A | hybrid |
| 162 | Chiusi _Lake | CytBhap5 | B | COIhap3 | B | N/A | RS |
| 45 | Po_River | N/A | N/A | N/A | N/A | 108 | N/A |
| 34 | Po_River | CytBhap6 | A | N/A | N/A | 106 | SS |
| 73 | Po_River | CytBhap6 | A | COIhap1 | A | 111 | SS |
| 61 | Po_River | CytBhap6 | A | COIhap1 | A | 110 | SS |
| 13 | Po_River | N/A | N/A | COIhap1 | A | 103 | SS |
| 74 | Po_River | CytBhap6 | A | COIhap1 | A | 111 | SS |
| 62 | Po_River | CytBhap6 | A | COIhap1 | A | 110 | SS |
| 46 | Po_River | CytBhap6 | A | COIhap1 | A | 108 | SS |
| 14 | Po_River | CytBhap6 | A | COIhap1 | A | 103 | SS |
| 75 | Po_River | N/A | N/A | COIhap5 | A | 111 | SS |
| 63 | Po_River | CytBhap6 | A | COIhap1 | A | 110 | SS |
| 53 | Po_River | N/A | N/A | N/A | N/A | 109 | N/A |
| 47 | Po_River | N/A | N/A | N/A | N/A | 108 | N/A |
| 35 | Po_River | CytBhap7 | A | COIhap1 | A | 106 | SS |
| 15 | Po_River | N/A | N/A | N/A | N/A | 103 | N/A |
| 64 | Po_River | N/A | N/A | COIhap1 | A | 110 | SS |
| 65 | Po_River | CytBhap6 | A | COIhap1 | A | 110 | SS |
| 21 | Po_River | CytBhap6 | A | COIhap1 | A | 104 | SS |
| 27 | Po_River | CytBhap6 | A | COIhap1 | A | 105 | SS |
| 163 | Danube_River | N/A | N/A | COIhap17 | A | N/A | RS |
| 96 | Danube_River | N/A | N/A | N/A | N/A | 136 | RS |
| 110 | Danube_River | N/A | N/A | COIhap20 | A | 140 | RS |
| 100 | Danube_River | N/A | N/A | N/A | N/A | 138 | N/A |
| 101 | Danube_River | N/A | N/A | N/A | N/A | 138 | N/A |
| 89 | Danube_River | N/A | N/A | N/A | N/A | 134 | N/A |
| 82 | Danube_River | N/A | N/A | N/A | N/A | 125 | RS |
| 84 | Danube_River | N/A | N/A | N/A | N/A | 128 | RS |
| 111 | Danube_River | N/A | N/A | COIhap19 | A | 140 | RS |
| 102 | Danube_River | N/A | N/A | N/A | N/A | 138 | N/A |
| 103 | Danube_River | N/A | N/A | N/A | N/A | 138 | N/A |
| 97 | Danube_River | N/A | N/A | COIhap18 | A | 136 | RS |
| 90 | Danube_River | N/A | N/A | N/A | N/A | 134 | N/A |
| 91 | Danube_River | N/A | N/A | N/A | N/A | 134 | N/A |
| 88 | Danube_River | N/A | N/A | N/A | N/A | 132 | N/A |
| 85 | Danube_River | N/A | N/A | N/A | N/A | 128 | RS |
| 54 | Maggiore_Lake | N/A | N/A | COIhap6 | B | 109 | RS |
| 16 | Maggiore_Lake | N/A | N/A | COIhap1 | A | 103 | SS |
| 66 | Maggiore_Lake | N/A | N/A | COIhap6 | B | 110 | LB |
| 3 | Maggiore_Lake | CytBhap2 | B | COIhap6 | B | 102 | RS |
| 22 | Maggiore_Lake | N/A | N/A | COIhap6 | B | 104 | RS |
| 17 | Maggiore_Lake | CytBhap3 | A | COIhap1 | A | 103 | LB |
| 4 | Maggiore_Lake | N/A | N/A | N/A | N/A | 102 | SS |
| 121 | Maggiore_Lake | CytBhap1 | B | COIhap6 | B | 147 | RS |
| 76 | Maggiore_Lake | N/A | N/A | N/A | N/A | 111 | SS |
| 28 | Bolsena_Lake | CytBhap3 | A | N/A | N/A | 105 | SS |
| 36 | Bolsena_Lake | N/A | N/A | N/A | N/A | 106 | SS |
| 77 | Bolsena_Lake | N/A | N/A | N/A | N/A | 111 | DB |
| 37 | Bolsena_Lake | CytBhap3 | A | COIhap1 | A | 106 | SS |
| 18 | Bolsena_Lake | CytBhap3 | A | COIhap1 | A | 103 | LB |
| 67 | Bolsena_Lake | N/A | N/A | N/A | N/A | 110 | DB |
| 1 | Bolsena_Lake | N/A | N/A | COIhap1 | A | 101 | LB |
| 68 | Bolsena_Lake | CytBhap3 | A | N/A | N/A | 110 | SS |
| 69 | Bolsena_Lake | N/A | N/A | N/A | N/A | 110 | SS |
| 40 | Bolsena_Lake | CytBhap3 | A | N/A | N/A | 107 | SS |
| 5 | Bolsena_Lake | N/A | N/A | COIhap1 | A | 102 | DB |
| 23 | Bolsena_Lake | N/A | N/A | COIhap1 | A | 104 | VB |
| 48 | Bolsena_Lake | N/A | N/A | N/A | N/A | 108 | VB |
| 49 | Bolsena_Lake | N/A | N/A | N/A | N/A | 108 | VB |
| 50 | Bolsena_Lake | CytBhap3 | A | COIhap7 | A | 108 | DB |
| 51 | Bolsena_Lake | N/A | N/A | COIhap1 | A | 108 | DB |
| 55 | Bolsena_Lake | N/A | N/A | COIhap1 | A | 109 | DB |
| 78 | Bolsena_Lake | N/A | N/A | COIhap1 | A | 111 | DB |
| 19 | Bolsena_Lake | N/A | N/A | N/A | N/A | 103 | SS |
| 24 | Bolsena_Lake | N/A | N/A | N/A | N/A | 104 | SS |
| 164 | Adda_River | N/A | N/A | N/A | N/A | N/A | DB |
| 165 | Adda_River | N/A | N/A | N/A | N/A | N/A | VB |
| 166 | Adda_River | N/A | N/A | N/A | N/A | N/A | VB |
| 167 | Adda_River | N/A | N/A | N/A | N/A | N/A | SS |
| 6 | Adda_River | N/A | N/A | N/A | N/A | 102 | LB |
| 7 | Adda_River | N/A | N/A | N/A | N/A | 102 | LB |
| 168 | Adda_River | N/A | N/A | N/A | N/A | N/A | RS |
| 169 | Adda_River | N/A | N/A | COIhap8 | A | N/A | N/A |
| 170 | Adda_River | N/A | N/A | COIhap3 | B | N/A | RS |
| 171 | Adda_River | CytBhap8 | A | COIhap4 | A | N/A | N/A |
| 172 | Adda_River | N/A | N/A | N/A | N/A | N/A | RS |
| 173 | Adda_River | N/A | N/A | COIhap6 | B | N/A | RS |
| 174 | Adda_River | N/A | N/A | N/A | N/A | N/A | RS |
| 2 | Adda_River | N/A | N/A | N/A | N/A | 101 | SS |
| 79 | Adda_River | N/A | N/A | N/A | N/A | 111 | SS |
| 8 | Adda_River | N/A | N/A | N/A | N/A | 102 | SS |
| 70 | Adda_River | N/A | N/A | N/A | N/A | 110 | SS |
| 118 | Danube_River | N/A | N/A | COIhap3 | B | 144 | RS |
| 119 | Danube_River | CytBhap9 | B | COIhap3 | B | 144 | RS |
| 112 | Danube_River | CytBhap10 | B | COIhap3 | B | 140 | RS |
| 114 | Danube_River | N/A | N/A | N/A | N/A | 142 | RS |
| 104 | Danube_River | CytBhap21 | B | COIhap6 | B | 138 | RS |
| 105 | Danube_River | N/A | N/A | COIhap6 | B | 138 | RS |
| 98 | Danube_River | CytBhap11 | B | COIhap3 | B | 136 | RS |
| 92 | Danube_River | CytBhap1 | B | COIhap6 | B | 134 | RS |
| 122 | Danube_River | CytBhap3 | A | COIhap6 | B | 148 | RS |
| 115 | Danube_River | N/A | N/A | N/A | N/A | 142 | RS |
| 120 | Danube_River | N/A | N/A | COIhap6 | B | 144 | RS |
| 175 | Danube_River | N/A | N/A | COIhap6 | B | N/A | RS |
| 176 | Danube_River | CytBhap2 | B | COIhap6 | B | N/A | RS |
| 117 | Danube_River | CytBhap1 | B | COIhap6 | B | 143 | RS |
| 177 | Danube_River | CytBhap12 | B | COIhap6 | B | N/A | RS |
| 116 | Danube_River | CytBhap1 | B | COIhap6 | B | 142 | RS |
| 178 | Danube_River | N/A | N/A | COIhap6 | B | N/A | RS |
| 179 | Danube_River | CytBhap4 | B | COIhap3 | B | N/A | RS |
| 180 | Danube_River | CytBhap4 | B | COIhap3 | B | N/A | RS |
| 181 | Danube_River | CytBhap5 | B | COIhap3 | B | N/A | RS |
| 182 | Danube_River | CytBhap13 | B | COIhap3 | B | N/A | RS |
| 183 | Danube_River | CytBhap5 | B | COIhap3 | B | N/A | RS |
| 184 | Chiusi_River | N/A | N/A | COIhap3 | B | N/A | RS |
| 185 | Chiusi_River | N/A | N/A | COIhap3 | B | N/A | RS |
| 186 | Chiusi_River | N/A | N/A | COIhap1 | A | N/A | SS |
| 187 | Chiusi_River | CytBhap4 | B | COIhap3 | B | N/A | RS |
| 188 | Chiusi_River | N/A | N/A | COIhap1 | A | N/A | N/A |
| 189 | Chiusi_River | N/A | N/A | COIhap11 | A | N/A | N/A |
| 190 | Chiusi_River | N/A | N/A | COIhap3 | B | N/A | N/A |
| 191 | Chiusi_River | CytBhap3 | A | COIhap4 | A | N/A | N/A |
| 192 | Chiusi_River | N/A | N/A | COIhap9 | A | N/A | N/A |
| 193 | Chiusi_River | CytBhap3 | A | COIhap1 | A | N/A | N/A |
| 194 | Chiusi_River | N/A | N/A | COIhap3 | B | N/A | RS |
| 195 | Chiusi_River | CytBhap3 | A | COIhap10 | A | N/A | N/A |
| 196 | Chiusi_River | CytBhap3 | A | COIhap4 | A | N/A | LB |
| 197 | Chiusi_River | CytBhap5 | B | COIhap3 | B | N/A | N/A |
| 198 | Chiusi_River | CytBhap3 | A | COIhap4 | A | N/A | SS |
| 199 | Chiusi_River | N/A | N/A | COIhap4 | A | N/A | SS |
| 200 | Chiusi_River | CytBhap3 | A | COIhap1 | A | N/A | SS |
| 25 | Trasimeno_Lake | N/A | N/A | N/A | N/A | 104 | DB |
| 201 | Piediluco | N/A | N/A | COIhap4 | A | N/A | SS |
| 202 | Piediluco | CytBhap3 | A | COIhap4 | A | N/A | SS |
| 203 | Piediluco | N/A | N/A | N/A | N/A | N/A | N/A |
| 204 | Piediluco | CytBhap3 | A | COIhap1 | A | N/A | hybrid |
| 205 | Piediluco | CytBhap10 | B | COIhap2 | B | N/A | RS |
| 206 | Piediluco | CytBhap3 | A | COIhap1 | A | N/A | SS |
| 29 | Trasimeno_Lake | N/A | N/A | COIhap1 | A | 105 | DB |
| 207 | Piediluco | CytBhap3 | A | COIhap4 | A | N/A | RS |
| 208 | Piediluco | CytBhap3 | A | COIhap1 | A | N/A | RS |
| 209 | Piediluco | CytBhap3 | A | COIhap1 | A | N/A | N/A |
| 210 | Piediluco | CytBhap3 | A | COIhap1 | A | N/A | N/A |
| 211 | Piediluco | N/A | N/A | COIhap1 | A | N/A | SS |
| 212 | Piediluco | CytBhap3 | A | N/A | N/A | N/A | DB |
| 213 | Piediluco | N/A | N/A | COIhap2 | B | N/A | hybrid |
| 214 | Piediluco | CytBhap3 | A | COIhap1 | A | N/A | DB |
| 215 | Piediluco | CytBhap3 | A | COIhap1 | A | N/A | DB |
| 216 | Piediluco | CytBhap3 | A | COIhap1 | A | N/A | SS |
| 217 | Piediluco | CytBhap3 | A | N/A | N/A | N/A | LB |
| 218 | Piediluco | N/A | N/A | N/A | N/A | N/A | SS |
| 219 | Piediluco | CytBhap11 | B | COIhap6 | B | N/A | RS |
| 220 | Piediluco | N/A | N/A | COIhap1 | A | N/A | SS |
| 221 | Piediluco | CytBhap14 | A | N/A | N/A | N/A | RS |
| 222 | Piediluco | N/A | N/A | COIhap4 | A | N/A | SS |
| 223 | Piediluco | CytBhap3 | A | COIhap1 | A | N/A | N/A |
| 224 | Segrino_Lake | CytBhap3 | A | COIhap1 | A | N/A | N/A |
| 225 | Segrino_Lake | CytBhap15 | A | COIhap1 | A | N/A | SS |
| 226 | Segrino_Lake | N/A | N/A | COIhap1 | A | N/A | SS |
| 227 | Segrino_Lake | CytBhap3 | A | COIhap1 | A | N/A | SS |
| 228 | Segrino_Lake | CytBhap3 | A | COIhap1 | A | N/A | SS |
| 229 | Segrino_Lake | CytBhap3 | A | N/A | N/A | N/A | SS |
| 230 | Segrino_Lake | N/A | N/A | N/A | N/A | N/A | SS |
| 231 | Segrino_Lake | N/A | N/A | N/A | N/A | N/A | SS |
| 232 | Segrino_Lake | N/A | N/A | N/A | N/A | N/A | LB |
| 233 | Segrino_Lake | N/A | N/A | N/A | N/A | N/A | SS |
| 234 | Segrino_Lake | N/A | N/A | N/A | N/A | N/A | LB |
| 235 | Garlate_Lake | N/A | N/A | N/A | N/A | N/A | N/A |
| 236 | Garlate_Lake | N/A | N/A | N/A | N/A | N/A | N/A |
| 237 | Garlate_Lake | CytBhap3 | A | COIhap1 | A | N/A | N/A |
| 238 | Garlate_Lake | N/A | N/A | N/A | N/A | N/A | N/A |
| 239 | Garlate_Lake | N/A | N/A | N/A | N/A | N/A | N/A |
| 240 | Garlate_Lake | N/A | N/A | COIhap1 | A | N/A | N/A |
| 241 | Garlate_Lake | N/A | N/A | N/A | N/A | N/A | N/A |
| 242 | Garlate_Lake | N/A | N/A | N/A | N/A | N/A | N/A |
| 243 | Garlate_Lake | N/A | N/A | N/A | N/A | N/A | N/A |
| 244 | Garlate_Lake | N/A | N/A | N/A | N/A | N/A | N/A |
| 245 | Garlate_Lake | N/A | N/A | N/A | N/A | N/A | N/A |
| 246 | Garlate_Lake | N/A | N/A | N/A | N/A | N/A | N/A |
| 247 | Garlate_Lake | N/A | N/A | N/A | N/A | N/A | N/A |
| 248 | Garlate_Lake | CytBhap3 | A | COIhap1 | A | N/A | N/A |
| 249 | Garlate_Lake | N/A | N/A | N/A | N/A | N/A | N/A |
| 368 | NederlaN/A | N/A | N/A | N/A | N/A | N/A | RS |
| 369 | NederlaN/A | N/A | N/A | N/A | N/A | N/A | RS |
| 370 | NederlaN/A | N/A | N/A | N/A | N/A | N/A | RS |
| 250 | Torbiere_Iseo | CytBhap16 | B | COIhap6 | B | N/A | N/A |
| 251 | Torbiere_Iseo | N/A | N/A | N/A | N/A | N/A | N/A |
| 252 | Torbiere_Iseo | N/A | N/A | COIhap1 | A | N/A | N/A |
| 253 | Torbiere_Iseo | N/A | N/A | COIhap1 | A | N/A | N/A |
| 254 | Torbiere_Iseo | N/A | N/A | COIhap4 | A | N/A | N/A |
| 255 | Trasimeno_Lake | N/A | N/A | COIhap1 | A | N/A | N/A |
| 256 | Trasimeno_Lake | CytBhap3 | A | COIhap1 | A | N/A | N/A |
| 257 | Trasimeno_Lake | N/A | N/A | COIhap1 | A | N/A | N/A |
| 258 | Trasimeno_Lake | CytBhap3 | A | COIhap1 | A | N/A | N/A |
| 259 | Trasimeno_Lake | CytBhap3 | A | COIhap1 | A | N/A | N/A |
| 260 | Trasimeno_Lake | CytBhap3 | A | COIhap1 | A | N/A | N/A |
| 261 | Brenta_River | CytBhap8 | A | COIhap13 | A | N/A | DB |
| 262 | Brenta_River | CytBhap19 | A | COIhap13 | A | N/A | DB |
| 263 | Brenta_River | CytBhap19 | A | COIhap13 | A | N/A | DB |
| 264 | Brenta_River | CytBhap19 | A | COIhap13 | A | N/A | DB |
| 265 | Brenta_River | CytBhap20 | A | N/A | N/A | N/A | DB |
| 266 | Brenta_River | CytBhap19 | A | COIhap13 | A | N/A | DB |
| 267 | Brenta_River | CytBhap19 | A | N/A | N/A | N/A | DB |
| 268 | Brenta_River | CytBhap20 | A | COIhap1 | A | N/A | DB |
| 270 | Brenta_River | CytBhap19 | A | N/A | N/A | N/A | DB |
| 271 | Brenta_River | CytBhap19 | A | COIhap13 | A | N/A | DB |
| 272 | Brenta_River | CytBhap19 | A | COIhap13 | A | N/A | DB |
| 273 | Brenta_River | CytBhap19 | A | COIhap13 | A | N/A | DB |
| 274 | Brenta_River | CytBhap19 | A | COIhap13 | A | N/A | DB |
| 275 | Brenta_River | CytBhap19 | A | COIhap13 | A | N/A | DB |
| 276 | Brenta_River | CytBhap19 | A | COIhap13 | A | N/A | DB |
| 277 | River_Sile | CytBhap19 | A | COIhap13 | A | N/A | DB |
| 278 | River_Sile | CytBhap19 | A | COIhap13 | A | N/A | DB |
| 279 | River_Sile | CytBhap19 | A | COIhap13 | A | N/A | DB |
| 280 | River_Sile | CytBhap19 | A | COIhap13 | A | N/A | DB |
| 281 | River_Sile | CytBhap19 | A | COIhap13 | A | N/A | DB |
| 282 | River_Sile | CytBhap3 | A | COIhap1 | A | N/A | RS |
| 283 | Fimon_Lake | CytBhap3 | A | COIhap1 | A | N/A | RS |
| 107 | Fimon_Lake | CytBhap1 | B | COIhap6 | B | 139 | RS |
| 284 | Fimon_Lake | CytBhap21 | B | COIhap6 | B | N/A | N/A |
| 285 | Fimon_Lake | CytBhap3 | A | N/A | N/A | N/A | RS |
| 286 | Fimon_Lake | CytBhap3 | A | N/A | N/A | N/A | RS |
| 287 | Fimon_Lake | CytBhap3 | A | N/A | N/A | N/A | RS |
| 288 | Fimon_Lake | CytBhap3 | A | N/A | N/A | N/A | RS |
| 289 | Fimon_Lake | CytBhap3 | A | N/A | N/A | N/A | DB |
| 99 | Sweden | CytBhap4 | B | COIhap14 | B | 136 | RS |
| 106 | Sweden | CytBhap1 | B | COIhap15 | B | 138 | RS |
| 83 | Sweden | N/A | N/A | COIhap15 | B | 127 | RS |
| 86 | Sweden | CytBhap1 | B | COIhap15 | B | 130 | RS |
| 108 | Sweden | CytBhap22 | B | COIhap14 | B | 139 | RS |
| 94 | Sweden | CytBhap1 | B | COIhap15 | B | 134 | RS |
| 87 | Sweden | CytBhap1 | B | COIhap6 | B | 130 | RS |
| 109 | Sweden | CytBhap1 | B | COIhap15 | B | 139 | RS |
| 95 | Sweden | CytBhap1 | B | COIhap15 | B | 134 | RS |
| 290 | Varese_Lake | CytBhap23 | B | N/A | N/A | N/A | N/A |
| 291 | Varese_Lake | CytBhap23 | B | N/A | N/A | N/A | N/A |
| 292 | Varese_Lake | CytBhap23 | B | N/A | N/A | N/A | N/A |
| 293 | Varese_Lake | CytBhap23 | B | N/A | N/A | N/A | N/A |
| 113 | Varese_Lake | CytBhap4 | B | COIhap3 | B | 140 | N/A |
| 294 | Varese_Lake | CytBhap23 | B | N/A | N/A | N/A | N/A |
| 295 | Varese_Lake | CytBhap4 | B | N/A | N/A | N/A | N/A |
| 296 | Varese_Lake | CytBhap23 | B | N/A | N/A | N/A | N/A |
| 297 | Po_Basin | CytBhap25 | A | N/A | N/A | N/A | N/A |
| 298 | Po_Basin | CytBhap24 | A | N/A | N/A | N/A | N/A |
| 299 | Po_Basin | CytBhap1 | B | N/A | N/A | N/A | N/A |
| 300 | Po_Basin | CytBhap3 | A | N/A | N/A | N/A | N/A |
| 301 | Po_Basin | CytBhap23 | B | N/A | N/A | N/A | N/A |
| 302 | Po_Basin | CytBhap3 | A | N/A | N/A | N/A | N/A |
| 303 | Po_Basin | CytBhap3 | A | N/A | N/A | N/A | N/A |
| 304 | Po_Basin | CytBhap3 | A | N/A | N/A | N/A | N/A |
| 305 | Po_Basin | CytBhap3 | A | N/A | N/A | N/A | N/A |
| 306 | Po_Basin | CytBhap3 | A | N/A | N/A | N/A | N/A |
| 307 | Po_Basin | CytBhap3 | A | N/A | N/A | N/A | N/A |
| 308 | Po_Basin | CytBhap3 | A | N/A | N/A | N/A | N/A |
| 309 | Po_Basin | CytBhap3 | A | N/A | N/A | N/A | N/A |
| 310 | Po_Basin | CytBhap3 | A | N/A | N/A | N/A | N/A |
| 311 | Po_Basin | CytBhap3 | A | N/A | N/A | N/A | N/A |
| 312 | Adige_River | CytBhap3 | A | N/A | N/A | N/A | DB |
| 313 | Adige_River | CytBhap3 | A | N/A | N/A | N/A | DB |
| 314 | Adige_River | CytBhap3 | A | N/A | N/A | N/A | DB |
| 315 | Adige_River | CytBhap3 | A | N/A | N/A | N/A | DB |
| 316 | Adige_River | CytBhap3 | A | N/A | N/A | N/A | DB |
| 317 | Corlo_Lake | CytBhap3 | A | N/A | N/A | N/A | RS |
| 318 | Corlo_Lake | CytBhap23 | B | COIhap6 | B | N/A | RS |
| 319 | Corlo_Lake | CytBhap23 | B | COIhap6 | B | N/A | RS |
| 320 | Corlo_Lake | CytBhap23 | B | COIhap6 | B | N/A | RS |
| 321 | Corlo_Lake | CytBhap23 | B | COIhap6 | B | N/A | RS |
| 322 | Corlo_Lake | N/A | N/A | COIhap6 | B | N/A | RS |
| 323 | Bacchiglione_River | CytBhap26 | B | COIhap6 | B | N/A | RS |
| 324 | Bacchiglione_River | N/A | N/A | N/A | N/A | N/A | DB |
| 325 | Bacchiglione_River | CytBhap8 | A | COIhap13 | A | N/A | hybrid |
| 326 | Bacchiglione_River | CytBhap8 | A | COIhap13 | A | N/A | DB |
| 327 | Bacchiglione_River | N/A | N/A | N/A | N/A | N/A | DB |
| 328 | Bacchiglione_River | N/A | N/A | N/A | N/A | N/A | DB |
| 329 | Bacchiglione_River | CytBhap8 | A | COIhap13 | A | N/A | DB |
| 330 | Bacchiglione_River | CytBhap8 | A | COIhap14 | B | N/A | N/A |
| 331 | Bacchiglione_River | CytBhap8 | A | COIhap13 | A | N/A | DB |
| 332 | Bacchiglione_River | CytBhap29 | A | COIhap13 | A | N/A | DB |
| 333 | Bacchiglione_River | CytBhap8 | A | COIhap13 | A | N/A | DB |
| 334 | Bacchiglione_River | N/A | N/A | COIhap13 | A | N/A | DB |
| 335 | Bacchiglione_River | CytBhap8 | A | COIhap13 | A | N/A | DB |
| 336 | Bacchiglione_River | CytBhap8 | A | COIhap13 | A | N/A | DB |
| 337 | Bacchiglione_River | CytBhap8 | A | COIhap13 | A | N/A | DB |
| 338 | Bacchiglione_River | CytBhap28 | A | COIhap13 | A | N/A | DB |
| 339 | Bacchiglione_River | CytBhap28 | A | COIhap13 | A | N/A | DB |
| 340 | Bacchiglione_River | CytBhap8 | A | COIhap13 | A | N/A | DB |
| 341 | Bacchiglione_River | CytBhap8 | A | COIhap13 | A | N/A | DB |
| 342 | River_Sile | CytBhap8 | A | COIhap13 | A | N/A | DB |
| 343 | River_Sile | CytBhap19 | A | COIhap13 | A | N/A | N/A |
| 344 | River_Sile | CytBhap19 | A | COIhap13 | A | N/A | SS |
| 345 | River_Sile | CytBhap3 | A | N/A | N/A | N/A | N/A |
| 346 | River_Sile | CytBhap19 | A | COIhap13 | A | N/A | N/A |
| 347 | River_Sile | CytBhap19 | A | COIhap13 | A | N/A | VB |
| 348 | River_Sile | CytBhap27 | A | COIhap16 | A | N/A | LB |
| 349 | River_Sile | CytBhap3 | A | N/A | N/A | N/A | VB |
| 350 | River_Sile | CytBhap3 | A | N/A | N/A | N/A | DB |
| 351 | River_Sile | CytBhap19 | A | COIhap13 | A | N/A | DB |
| 352 | River_Sile | CytBhap3 | A | N/A | N/A | N/A | SS |
| 353 | River_Sile | CytBhap3 | A | N/A | N/A | N/A | SS |
| 354 | River_Sile | CytBhap3 | A | N/A | N/A | N/A | SS |
| 355 | River_Sile | CytBhap3 | A | N/A | N/A | N/A | DB |
| 356 | River_Sile | CytBhap3 | A | N/A | N/A | N/A | DB |
| 357 | Po_Veneto | CytBhap19 | A | COIhap13 | A | N/A | DB |
| 358 | Po_Veneto | CytBhap6 | A | N/A | N/A | N/A | LB |
| 359 | Po_Veneto | CytBhap21 | B | N/A | N/A | N/A | hybrid |
| 360 | Po_Veneto | CytBhap3 | A | N/A | N/A | N/A | LB |
| 361 | Po_Veneto | CytBhap30 | A | N/A | N/A | N/A | SS |
| 362 | Po_Veneto | CytBhap6 | A | N/A | N/A | N/A | RS |
| 363 | Po_Veneto | CytBhap3 | A | N/A | N/A | N/A | LB |
| 364 | Po_Veneto | CytBhap3 | A | N/A | N/A | N/A | DB |
| 365 | Po_Veneto | CytBhap3 | A | N/A | N/A | N/A | SS |
| 366 | Po_Veneto | CytBhap6 | A | N/A | N/A | N/A | RS |
| 367 | Po_Veneto | CytBhap3 | A | N/A | N/A | N/A | SS |
| 371 | SwitzerlaN/A | N/A | N/A | N/A | N/A | N/A | RS |
| 372 | SwitzerlaN/A | N/A | N/A | N/A | N/A | N/A | RS |
| 373 | SwitzerlaN/A | N/A | N/A | N/A | N/A | N/A | RS |
| 374 | SwitzerlaN/A | N/A | N/A | N/A | N/A | N/A | RS |
