# Supplementary material for: Molecular and Phenotypic Evidence of a New Species of Genus Esox (Esocidae, Esociformes, Actinopterygii): The Southern Pike, Esox flaviae
Source: PLoS One. 2011 Dec 2;6(12):e25218. doi: 10.1371/journal.pone.0025218 (PMC3229480; doi:10.1371/journal.pone.0025218)
Supplement: Text S4 — Permission to publish Figure 1 –Monday, July 11th, 2011 17.08. (DOC) [file pone.0025218.s004.doc]

**SUPPLEMENTARY TEXT S4**

Permission to publish Figure 1 - Monday, July 11^th^, 2011 17.08

Monday, July 11^th^, 2011 17.08

**Subject:** Molecular and Phenotypic Evidence of a New Species of Genus *Esox* (Esocidae, Esociformes, Actinopterygii): the Southern Pike, *Esox flaviae*

We grant permission for submission entitled "Molecular and Phenotypic Evidence of a New Species of Genus *Esox* (Esocidae, Esociformes, Actinopterygii): the Southern Pike, *Esox* *flaviae*" to Lucentini et alii in PLOS ONE, to reproduce the Stefani paintings providing you acknowledge the source and  that the utilisation of our materials on your part is no-profit.

Yours faithfully

Settore Ufficio Stampa e Comunicazione
Il Dirigente
Dr. Domenico Vita
Veneto Agricoltura
V.le dell'Università 14
35020 Legnaro (PD)
Tel. 049-8293768  Fax 8293754
